# Meta-omics profiling of the gut-lung axis illuminates metabolic networks and host-microbial interactions associated with elevated lung elastance in a murine model of obese allergic asthma

**DOI:** 10.3389/frmbi.2023.1153691

**Published:** 2023-05-05

**Authors:** Victoria A. Heinrich, Crystal Uvalle, Michelle L. Manni, Kelvin Li, Steven J. Mullett, Sri Ramya Donepudi, Jason Clader, Adam Fitch, Madeline Ellgass, Veronika Cechova, Shulin Qin, Fernando Holguin, Bruce A. Freeman, Barbara A. Methé, Alison Morris, Stacy L. Gelhaus

**Affiliations:** 1 Department of Pharmacology and Chemical Biology, School of Medicine, University of Pittsburgh, Pittsburgh, PA, United States,; 2 Medical Scientist Training Program, School of Medicine, University of Pittsburgh, Pittsburgh, PA, United States,; 3 Health Sciences Mass Spectrometry Core, University of Pittsburgh, Pittsburgh, PA, United States,; 4 Center for Medicine and the Microbiome, University of Pittsburgh, Pittsburgh, PA, United States,; 5 Integrative Systems Biology Program, School of Medicine, University of Pittsburgh, Pittsburgh, PA, United States,; 6 Division of Pulmonary, Allergy and Critical Care Medicine, Department of Medicine, University of Pittsburgh, Pittsburgh, PA, United States,; 7 Division of Pulmonary Sciences and Critical Care, School of Medicine, University of Colorado Anschutz Medical Campus, Aurora, CO, United States,; 8 Department of Clinical and Translational Science, University of Pittsburgh, Pittsburgh, PA, United States

**Keywords:** asthma, microbiome, obesity, metabolomics, multi-omics analyses, elastance, proline, gut-lung axis

## Abstract

Obesity and associated changes to the gut microbiome worsen airway inflammation and hyperresponsiveness in asthma. Obesogenic host-microbial metabolomes have altered production of metabolites that may influence lung function and inflammatory responses in asthma. To understand the interplay of the gut microbiome, metabolism, and host inflammation in obesity-associated asthma, we used a multi-omics approach to profile the gut-lung axis in the setting of allergic airway disease and diet-induced obesity. We evaluated an immunomodulator, nitro-oleic acid (NO_2_-OA), as a host- and microbial-targeted treatment intervention for obesity-associated allergic asthma. Allergic airway disease was induced using house dust mite and cholera toxin adjuvant in C57BL6/J mice with diet-induced obesity to model obesity-associated asthma. Lung function was measured by flexiVent following a week of NO_2_-OA treatment and allergen challenge. 16S rRNA gene (from DNA, taxa presence) and 16S rRNA (from RNA, taxa activity) sequencing, metabolomics, and host gene expression were paired with a Treatment-Measured-Response model as a data integration framework for identifying latent/hidden relationships with linear regression among variables identified from high-dimensional meta-omics datasets. Targeting both the host and gut microbiota, NO_2_-OA attenuated airway inflammation, improved lung elastance, and modified the gut microbiome. Meta-omics data integration and modeling determined that gut-associated inflammation, metabolites, and functionally active gut microbiota were linked to lung function outcomes. Using Treatment-Measured-Response modeling and meta-omics profiling of the gut-lung axis, we uncovered a previously hidden network of interactions between gut levels of amino acid metabolites involved in elastin and collagen synthesis, gut microbiota, NO_2_-OA, and lung elastance. Further targeted metabolomics analyses revealed that obese mice with allergic airway disease had higher levels of proline and hydroxyproline in the lungs. NO_2_-OA treatment reduced proline biosynthesis by downregulation of pyrroline-5-carboxylate reductase 1 (PYCR1) expression. These findings are relevant to human disease: adults with mild-moderate asthma and BMI ≥ 25 had higher plasma hydroxyproline levels. Our results suggest that changes to structural proteins in the lung airways and parenchyma may contribute to heightened lung elastance and serve as a potential therapeutic target for obese allergic asthma.

## 1 Introduction

As many as half of U.S. adults with metabolically unhealthy obesity also have asthma (Greiner and Hartwell, 2022). Obese individuals (body mass index, BMI, > 30 kg/m^2^) make up the majority of adults with severe asthma (Schatz et al., 2014). Patients with obesity-associated asthma experience poor asthma control and increased hospitalization risk (Mosen et al., 2008; Greiner and Hartwell, 2022). While weight gain and obesity carry an increased risk of developing airway hyperresponsiveness (Litonjua et al., 2002; Sharma et al., 2008), treating obesity with bariatric surgery improves asthma control and airway hyperresponsiveness (Dixon et al., 2011). The connection between obesity and asthma extends to the gut microbiome. In murine models, an obese gut microbiome phenotype enhances ozone-induced airway hyperresponsiveness while depleting an obese gut microbiome with antibiotics alleviates airway hyperresponsiveness (Cho et al., 2018; Tashiro et al., 2019). Diet-induced obesity drives loss of microbial diversity and beneficial microbial taxa known to attenuate inflammation and regulate host metabolism (Ley et al., 2006; Turnbaugh et al., 2006; Schneeberger et al., 2015). Obesity and an obesity-altered gut microbiome exacerbate airway reactivity and resistance, resulting in worse disease control and lung function.

The molecular mechanisms responsible for worse outcomes in obesity-associated asthma are still not well-understood, and targeted therapies for the treatment of obesity-associated asthma are needed. Small molecule nitroalkenes such as nitro-oleic acid (NO_2_-OA) show promise for treating metabolic diseases with systemic inflammation, including obesity-associated asthma (Kelley et al., 2014; Schopfer et al., 2018; Khoo et al., 2019). As NO_2_-OA is predominantly absorbed in the gut and dampens inflammation in the large bowel where the majority of the gut microbiota reside (Borniquel et al., 2010; Fazzari et al., 2017 NO_2_-OA may positively impact the gut microbiome as well.

We hypothesized that NO_2_-OA treatment improves lung function and modifies gut microbiota composition. Here we demonstrate that NO_2_-OA targets both host signaling and the gut microbiota and has potential as a treatment for obesity-associated asthma. We measured lung function in a murine model of obese allergic asthma after oral NO_2_-OA administration and characterized the gut microbiome with 16S rRNA gene sequencing. We then deployed a meta-omics approach to profiling the gut-lung axis. This allowed us to define metabolic networks and host-microbial interactions contributing to potential molecular mechanisms of obese allergic asthma.

## 2 Materials and methods

### 2.1 Human studies

All human studies received approval from the Institutional Review Board of the University of Pittsburgh in accordance with The Code of Ethics of the World Medical Association. Study participants were enrolled through the Electrophilic Fatty Acid Derivatives in Asthma study at Pittsburgh (PRO11010186). Male and female participants were nonsmokers in the last year and had a 10 or less pack-year smoking history. Participants had mild-moderate asthma with a forced expiratory volume in 1 second (FEV1) greater than 60% of predicted value and were taking either no controller medications or up to low- to moderate-dose inhaled corticosteroids (ICS) with or without a second controller agent (leukotriene modifier or long-acting β-agonist) as clinically indicated. Per mandatory reporting requirements by the NIH Inclusion of Women, Minorities, and Children policy, study participants self-reported race from racial categories defined by the NIH Office of Management and Budget (OMB) Revisions to the Standards for the Classification of Federal Data on Race and Ethnicity. Of the study participants with mild-moderate asthma, 57% self-reported as White, and 43% self-reported as Black. Blood draws, fraction of exhaled nitric oxide (FeNO) measurement, and baseline and postbronchodilator spirometry following ATS guidelines were completed (Moore et al., 2011; Fajt et al., 2013). Plasma was obtained for metabolomics analysis by liquid chromatography-high resolution mass spectrometry (LC-HRMS).

### 2.2 Murine model of diet-induced obesity and allergic airway disease

4-week-old, male C57BL/6J mice (000664, Jackson Laboratory, Bar Harbor, ME) were fed high fat diet (60% kcal fat diet D12492, Research Diets, Inc., New Brunswick, NJ) for 15 weeks. During allergic airway disease induction, which mice were sensitized to 2 μg house dust mite (*D. pteronyssinus*, Greer Lot #213051, endotoxin level 32.25 EU/vial, Greer Laboratories Inc, Lenoir, NC) with 0.1 μg cholera toxin (from *Vibrio cholera*, Lot #10070A1, List Biological Laboratories Inc, Campbell, CA) adjuvant *via* oropharyngeal aspiration. Mice were treated with either glycerol trioleate (Sigma Aldrich, St. Louis, MO) vehicle (AAD-Vehicle group, n = 11) or 9,10-nitro-oleic acid, NO_2_-OA, (AAD-NO_2_-OA group, n = 11) *via* gavage, followed by challenge 3 hours later with 2 μg house dust mite antigen *via* oropharyngeal aspiration. Controls for obese mice with allergic airway disease included obese mice without allergic airway disease that only received adjuvant during sensitization and house dust mite (HDM) during the challenge (Control group, n = 12) and a group of naïve obese mice (Naïve group, n = 7). The CT, HDM or treatments did not alter animal weight (Supplement Figure 1). All mouse experiments were conducted in accordance with NIH ARRIVE guidelines and approved by the University of Pittsburgh IACUC (Protocol #20016689).

### 2.3 Lung function measurement

Mice were anesthetized with an intraperitoneal injection of 100 mg/kg pentobarbital, and a surgical tracheotomy with a cannula was performed. Mice were subsequently mechanically ventilated at 150 breaths/min with a tidal volume of 10 mL/kg and a positive end expiratory pressure of 3 cmH_2_O using a computer-controller small-animal ventilator (flexiVent, SCIREQ, Montreal, Quebec, Canada). Baseline lung function was recorded using pressure-volume curves to measure the quasi-static lung parameters of compliance and hysteresis. An integrated nebulizer delivered 0, 3.125, 12.5, 25, and 50 mg/mL methacholine aerosol challenges to the mouse lungs, and respiratory mechanics were assessed using the single compartment (single frequency forced oscillations) and constant phase (multiple frequency forced oscillations) models to generate dose-response curves. Using Flexiware 8.2.0 software (SCIREQ, Montreal, Quebec, Canada), measured pressure and volume datasets for each individual mouse were fit with multiple linear regressions, from which Newtonian resistance (R_n_), tissue damping (G), tissue elastance (H), total respiratory resistance (R_rs_), and total respiratory elastance (E_rs_) were calculated.

### 2.4 Bronchoalveolar lavage fluid, serum, and tissue collection

After lung function measurement, bronchoalveolar lavage fluid (BALF) was collected by flushing the lungs with 1 mL phosphate buffered saline (Gibco PBS, pH = 7.4, Thermo Fisher Scientific, Waltham, MA) through the tracheal cannula. BALF was transferred to a 10 mL glass centrifuge tube and centrifuged at 1500 rpm at 4°C for 10 min to pellet BAL immune cells. BALF supernatant was removed and stored in a new 10 mL glass centrifuge tube at −80°C until further downstream processing. Pelleted BAL immune cells were reconstituted in 500 μL phosphate buffered saline (Gibco PBS, pH = 7.4, Thermo Fisher Scientific) and counted as previously described (Manni et al., 2014; Manni et al., 2016). Blood was collected *via* cardiac puncture and transferred to serum separator tubes on ice. Serum separator tubes were thawed at room temperature for 30 min and then centrifuged at 10,000 × g for 90 seconds. Murine lung, cecum, and mid-colon tissue were collected immediately after sacrifice and flash-frozen with liquid nitrogen. All BALF, serum, lung, cecum, and mid-colon tissue samples were stored at −80°C until further downstream processing.

### 2.5 Cytokine and chemokine measurement

30–60 mg of crushed lung tissue was weighed out into 1.5 mL Eppendorf Safe-Lock Tubes (Eppendorf AG #022363212, Hamburg, Germany). To completely homogenize lung tissue, crushed lung samples were sonicated in 1 mL PBS (Gibco #10010023, pH = 7.4, Thermo Fisher Scientific) buffer with PhosSTOP (Sigma Aldrich #4906837001, St. Louis, MO) and cOmplete ULTRA EDTA-free (Sigma Aldrich #5892970001) protease inhibitors. Cytokine and chemokine concentrations normalized to protein amount were measured in lung homogenates with a Bio-Plex Pro Mouse Cytokine 23-plex Assay (Bio-Rad Laboratories, Hercules, CA, #M60009RDPD) and the Bio-Plex 200 suspension array system (Bio-Rad) following the manufacturer’s instructions.

### 2.6 Murine tissue gene expression with real-time quantitative polymerase chain reaction

mRNA gene expression for the gut and lungs was measured in RNA extracted from mid-colon tissue and crushed lung tissue, respectively. Flash-frozen murine lungs were crushed on ice with mortar and pestle. Mid-colon tissue was rinsed with PBS (Gibco PBS, pH = 7.4, ThermoFisher Scientific) to remove stool contents and homogenized in 2 mL lysing tubes (Lysing Matrix A, MP Biomedicals, Solon, OH) with an automated tissue homogenizer (FastPrep-24 5G Homogenizer, MP Biomedicals). RNA was extracted from crushed lung and bead-homogenized mid-colon tissue with Thermo Fisher TRIZol reagent following the manufacturer’s instructions. cDNA was synthesized from 1 μg template RNA with the iScript cDNA synthesis kit (Bio-Rad Laboratories, Hercules, CA). mRNA expression was measured by RT-qPCR using TaqMan Fast Advanced Master Mix (Applied Biosystems, Waltham, MA) and TaqMan Gene Expression Assays (Applied Biosystems) with FAM-dye labeled gene primers and VIC-dye labeled mouse *Gapdh* (4352339E, Applied Biosystems) as the housekeeping gene. FAM-dye labeled primers (Applied Biosystems) were used for the following target genes in the lungs: *Ano1* (Mm00724407_m1), *Ccl8* (Mm01297183_m1), *Clca1* (Mm01320697_m1), *Cxcl1* (Mm04207460_m1), *Cxcl15* (Mm04208136_m1), *IL6* (Mm00446190_m1), *Lox* (Mm00495386_m1), *Muc5ac* (Mm01276718_m1), *Muc5b* (Mm00466391_m1), *Nos2* (Mm00440502_m1), *P4ha1* (Mm00803137_m1), *Pparg* (Mm01184322_m1), *Prg2* (Mm00435905_m1), and *Pycr1* (Mm00522678_m1). FAM-dye labeled primers (Applied Biosystems) were used for the following target genes in the colon: *Cox2* (Mm03294838_g1), *IL6* (Mm00446190_m1), *Nos2* (Mm00440502_m1), *Pparg* (Mm01184322_m1), and *Tnf* (Mm00443258_m1). RT-qPCR reactions were prepared following manufacturer instructions and amplified for 40 cycles with the Applied Biosystems StepOne Real-Time PCR system. Gene expression was quantified using the 2^−ΔΔCt^ method (Livak and Schmittgen, 2001).

### 2.7 Metabolomics with liquid chromatography-high resolution mass spectrometry

Amino acids (including hydroxyproline), organic acids and other metabolites were measured by untargeted LC-HRMS in murine serum, stool, cecum, and lung tissue. Proline and hydroxyproline were also measured in the plasma of adults with mild-moderate asthma by untargeted LC-HRMS.

For LC-HRMS sample preparation, metabolic quenching and polar metabolite pool extraction was performed by adding ice cold 80% methanol (aqueous) at a ratio of 1:15 (1 mg: 15 μL) for tissue and through the addition of ice cold 1:1 methanol:ethanol at a ratio of 1:4 serum/plasma:solvent. ^13^C-creatinine, taurine-_d4_, lactate-_d3_ and alanine-_d3_ (Sigma-Aldrich) were added to the sample lysates as an internal standard at a final concentration of 10 μM. Tissue samples were homogenized using a MP Bio FastPrep system using Matrix D (ceramic sphere) for 60 seconds at 60 Hz. The tissue and serum supernatants were then cleared of protein by centrifugation at 16,000 × g.

For analyses performed by untargeted LC-HRMS, cleared supernatant (2 μL) was subjected for online analysis *via* a Thermo Vanquish UHPLC and separated over a reversed phase Thermo HyperCarb porous graphite column (2.1×100 mm, 3μm particle size) maintained at 55°C. For the 20-minute LC gradient, the mobile phase consisted of the following: solvent A (water/0.1% formic acid, FA) and solvent B (acetonitrile, ACN/0.1% FA). The gradient was the following: 0–1min 1% B, with an increase to 15%B over 5 min, increasing to 98%B over 5 min, holding at 98%B for 5 min, and then equilibration at 1%B for 5 min. The Thermo ID-X tribrid mass spectrometer was operated in both positive and negative ion mode, scanning in ddMS^2^ mode (2 μscans) from 70 to 800 *m/z* at 120,000 resolution with an automatic gain control (AGC) target of 2e5 for full scan, 2e4 for MS^2^ scans using high-energy collisional dissociation (HCD) fragmentation at stepped 15, 35, 50 collision energies. Source ionization settings were 3.0 and 2.4kV spray voltage for positive and negative mode, respectively. Source gas parameters were 35 sheath gas, 12 auxiliary gas at 320°C, and 8 sweep gas. Calibration was performed prior to analysis using the PierceTM FlexMix Ion Calibration Solutions (Thermo Fisher Scientific). Integrated peak areas were then extracted manually using Quan Browser (Thermo Fisher Xcalibur ver. 2.7). Data is reported as the Relative Amount, which is the peak area ratio of the analyte normalized to the internal standard.

### 2.8 16S rRNA gene and 16S rRNA sequencing

Passed murine stool was collected with sterile technique prior to lung function measurement. Stool RNA and DNA were isolated using ZymoBIOMICS DNA/RNA Miniprep kit (Zymo Research Corporation, Irvine, CA). cDNA was synthesized (SuperScript IV VILO kit, ThermoFisher Scientific, Waltham, MA). 16S rRNA amplicons were run on Illumina MiSeq (Illumina, San Diego, CA) (Caporaso et al., 2012; Stapleton et al., 2021). 16S rRNA reads were processed for quality control with an in-house pipeline; taxonomic profiles adjusted for compositional data with the additive log ratio transformation were generated (Wang et al., 2007; Cole et al., 2009; Schloss et al., 2009; Caporaso et al., 2012; Quast et al., 2013; Tarabichi et al., 2015). Additional details on the 16S rRNA gene and 16S rRNA sequencing and processing are included in the Supplemental Information. Regression modeling using transformed taxonomic abundances was performed. We fitted multiple linear regression models using ALR transformed taxonomic abundances as predictors (X’s) and lung function parameters as responses (Y’s) to associate changes in gut microbiota composition with pulmonary function in obese mice with asthma. We first applied the ALR transformation to relative abundances, so that the resultant transformed abundances could be analyzed as independent and normally distributed values (Tarabichi et al., 2015). Two linear models (Y=X, multivariate in Y, multiple in X, regression) were fitted and evaluated. The first model assumed that the microbiome (X) was a predictor of lung function parameters (Y), then a second model assumed that the lung function parameters were predictors (X) of the microbiome (Y). For assessment of beta-diversity and microbial community composition similarity/dissimilarity, multidimensional scaling (MDS) plots based on Euclidean inter-sample distances were calculated for the 16S rRNA ALR-transformed taxonomic abundances assessed through gene sequencing data. Permutational multivariate analysis of variance (PERMANOVA) was performed to test the association of 16S composition with covariates.

### 2.9 Meta-omics data integration and analysis with treatment-measured-response framework

The Treatment-Measured-Response (TMR) framework was devised to provide a systematic approach for testing the relationships among groups of related variables with linear modeling. Measurements of high dimensional “-omics” datasets benefit from their analysis as a group since they typically represent a particular biological compartment, where there may be a high degree of within group variable correlation. Within group correlations are of less interest in the overall TMR framework, since they may represent components of a pathway or cascade of inseparable events within a compartment. However, between group associations provide a means to integrate –omics datasets because they may reflect the bridges of inter-compartmental signaling. In the TMR modeling, variable groups (e.g. 16S rRNA, 16S rRNA gene, host lung and colon gene expression, lung, serum and cecum metabolomics, HDM, NO_2_-OA and high fat diet treatment, flexiVent measurements, etc.) are assigned to one of the three framework groupings. The “Treatment” group includes covariates, which cumulatively represent variables that were under control of the experimenter (treatments) or are essentially fixed (sex, age, etc.) during the experiment. Weight was chosen to represent the high fat diet treatment and was included in the model as percentage increase and final weight. The “Response” variables included the flexiVent measurements (baseline and slopes), which represents the clinical manifestation of the studied disease. Treatment and Response grouped variables are always considered predictors (x) and response (y) variables, respectively, in the TMR framework. The – omics datasets are analyzed in the “Measured” group. The variables in the “Measured” group may be predicted by the “Treatment” group and/or may be predictors of the variables in the “Response” group. The variables in the “Measured” group may also be predictors or responders to variables in other “Measured” groups. The following categories of linear models are fit:

1. Effect of Treatment (x) on Measured (y):
Measured[i] = Treatment
2. Effect of Measured (x) on Measured (y), controlling for Treatment (x). Where i and j are different datasets. Two models are explicitly stated to emphasize that the members of each pair of measured variables will participate as a predictor and then as a response in the two separate linear models:
Measured[i] = Measured[j] + Treatment
Measured[j] = Measured[i] + Treatment
3. Effect of Measured (x) on Response (y), controlling for Treatment (x):
Response = Measured[i] + Treatment

Note that in the above models, for clarity, only one treatment and response group was illustrated, but multiple treatment and response groups may be included and tested separately in the same TMR framework. In general, and as was implemented by including the suite of flexiVent measurements, if the Response group is specified as a group of continuous variables (in contrast to a single Boolean outcome or disease state) then the TMR framework has the flexibility to associate specific variables from the Measured groups to specific aspects of disease manifestation. After the regression models have been fit and coefficient and p-values have been estimated, network figures are generated at various p-value cutoffs to illustrate the relationship across the variables in the groups by connecting statistically significant associations between predictors and response variables with lines. If there is a significant bi-directional association between two variables from two Measured groups, then the stronger association (direction) is selected. Supplement Table 1 contains a summary of the most significant associations between the grouped variables. Note that in some cases, the predictor variable is a member of the treatment/covariate group and the model column indicates that a measured group is the predictor. This indicates that the treatment/covariate variable’s association with the response variable was significant when the measured group’s variables were included (controlled for) in the model.

In contrast to the networks built with pair-wise correlations calculated between all variables, the relationships identified by the TMR framework is driven by and restricted to the relationships determined by the experiment’s design. Covariates may be controlled for when multi-variable linear regression models identify associations between predictors and response variables. In addition, in multi-omic datasets, the number of biomarker variables in each dataset can be substantial, however Principal Component Analyses (PCAs) frequently reveal that only a small proportion of the Principal Components (PCs) are necessary to capture the majority of the variance in a dataset. The strategy for using PCA as a means to select variables for inclusion into the TMR calculations is described in the following Section 2.10.

In addition to identifying variable specific relationships between TMR groupings, a dendrogram of variable groupings was calculated based on the inter-sample distances estimated for each variable grouping with Mantel’s test statistic (Mantel, 1967). Using only the variables in each variable grouping, a Euclidean distance matrix (N × N) is calculated between all the samples. For every pair of variable groupings, the Pearson’s correlation coefficient is calculated between their respective distance matrices in the manner of Mantel’s test statistic for comparing distance matrices. The distance between groupings is then defined as the 1-|cor(x,y)|, where x and y are the distance matrices and cor is the Mantel’s test statistic. This intergroup distance is then used to hierarchically cluster the variable grouping types with Ward’s minimum variance algorithm and the dendrogram can be generated. As a result, variable grouping that are the most closely related to each other, cluster together in the dendrogram. These distance-based results should support the TMR regression-based relationships such that variable groups that are the most connected between their variables will also cluster together in the dendrogram.

### 2.10 Principal component analysis-based variable selection

Variable selection, for inclusion into the TMR framework, was performed in two steps for each meta-omics dataset, independently: 1.) Perform PCA to identify the number of principal components (PCs) necessary to sufficiently represent the variance in the dataset. 2.) Identify the closest representing variables in the dataset to represent the identified PCs. The goals of variable selection were to reduce the number variables included in the framework and ensure both that the variables that were selected would cumulatively represent a significant proportion of the variance (information) and not be highly correlated with one another. The steps included in the variable selection process are the following: First, variables were tested with the Shapiro-Wilks test to determine if they are normally distributed. If a variable is not normally distributed, then both a square root and logarithm transform are applied, separately. If either transformation improved the normality of the variable, then the better transformation is accepted. PCA was then performed on the correlation matrix calculated across the (transformed, if necessary) variables. Only PCs that could contribute more than 1% of the total variance in the dataset were retained. For each of the retained PCs, the correlation with each of the underlying (transformed) variables in the dataset (that were utilized in the PCA) was calculated, and an underlying variable was selected as a proxy for each of the PCs. Across our datasets, fewer than 10 PC variables were selected per dataset, while still accounting for more than 85% of the variance in each dataset. The selected underlying variables that are used as proxies for each of the selected PCs tended to have correlations >0.90, thus making them viable representatives. This provides a means for the interested reader to also examine the unselected variables and their relationship to each PC. Note, for the two 16S datasets, only the top 10 taxa (by average abundance) were included. This is a reasonable, unbiased approach because the top taxa account for the majority of taxonomic abundances, and the lower abundance taxa may have greater coefficients of variance.

### 2.11 Statistical analysis

All analyses, with the exception of the Treatment-Measured-Response model (which was implemented with custom code in R and using adonis2 from the vegan library), used GraphPad Prism 9 (GraphPad Software, San Diego, CA). Dose response curves were analyzed using two-way analysis of variance (ANOVA) with Tukey’s multiple comparisons test. One-way ANOVA with Tukey’s multiple comparisons test or one-way Welch’s ANOVA for unequal variance with Dunnett’s multiple comparisons were performed for experiments with one variable. Outliers were identified using the ROUT method (Q = 1%).

## 3 Results

### 3.1 NO_2_-OA reduced total inflammatory cells present in the lung airspaces

**FIGURE 1:**
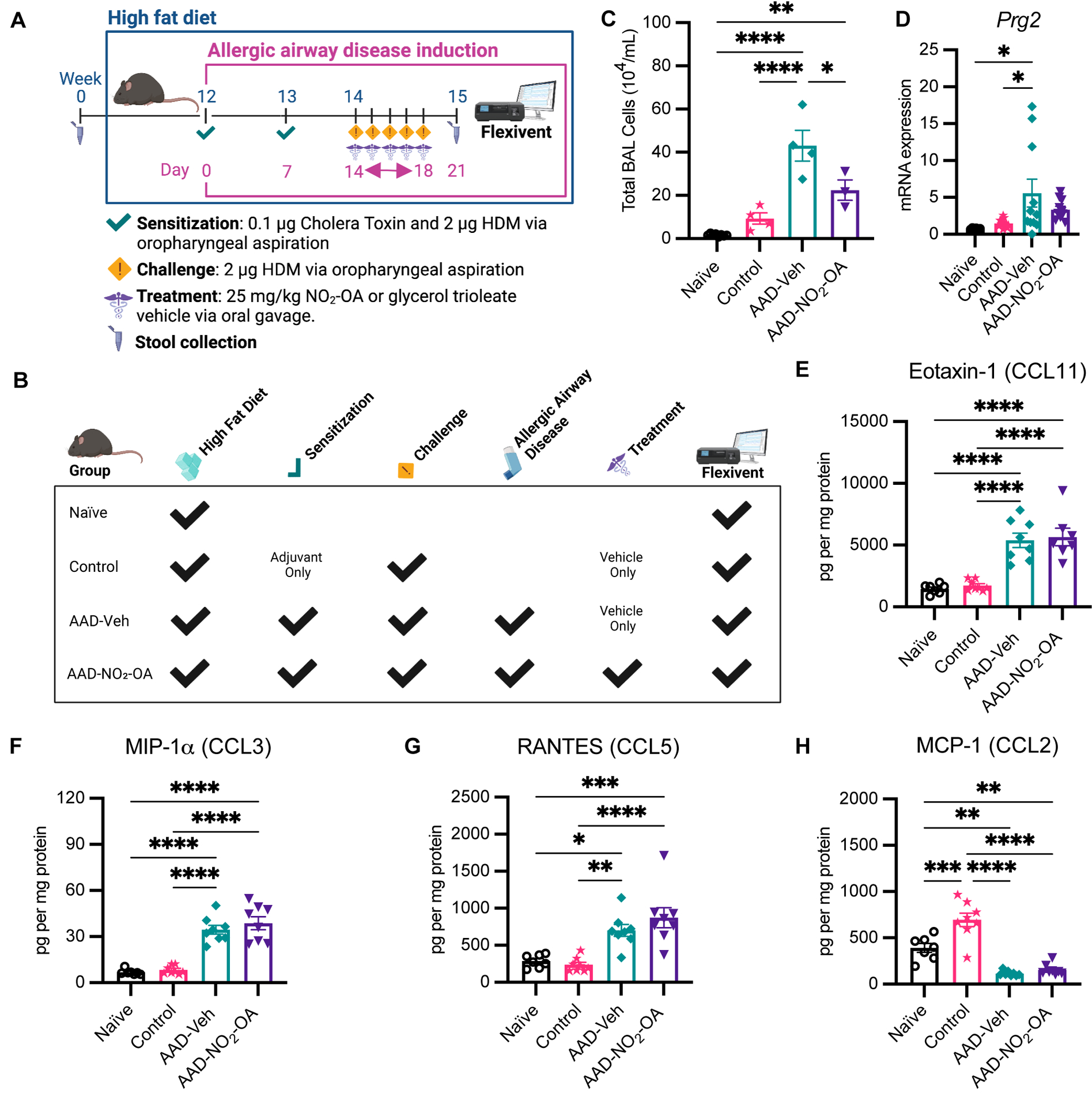
NO_2_-OA reduced total inflammatory cells present in the lung airspaces. **(A)** Murine model of obese allergic asthma in which C57BL/6J male mice developed diet-induced obesity and allergic airway disease with sensitization to house dust mite (HDM) and cholera toxin adjuvant, followed by daily treatment and HDM challenge *via* the oropharyngeal route. **(B)** Schematic of treatments received by each group. **(C)** Total inflammatory cells in the airspaces were measured in the bronchoalveolar lavage (BAL) fluid as 10^4^ cells per mL BAL fluid. Treatment groups included obese naïve (Naïve, n = 8), mock sensitization control (Control, n = 4), AAD-Vehicle (AAD-Veh, n = 4), and AAD-NO_2_-OA (n = 3). **(D)** Lung mRNA expression of eosinophil granule protein proteoglycan 2, *Prg2*, was measured with RT-qPCR normalized to *Gapdh*. **(E)** Eotaxin-1 (CCL11), **(F)** MIP-1α (CCL3), **(G)** RANTES (CCL5), and **(H)** MCP-1 (CCL2) cytokine protein levels normalized to mg protein were measured in lung homogenates with multiplex immunoassays. Treatment groups included obese naïve (Naïve, n = 7), mock sensitization control (Control, n = 12), AAD-Vehicle (AAD-Veh, n = 11), and AAD- NO_2_-OA treatment (n =11). Values are shown as mean ± SEM. Statistical significance was calculated by ordinary one-way ANOVA with Tukey’s multiple comparisons test, *p< 0.05, **p< 0.01, ***p< 0.001, ****p< 0.0001.

Obesity-associated allergic asthma was modeled by murine diet-induced obesity and allergic airway disease (AAD) using HDM as an allergic antigen and Cholera Toxin (CT) adjuvant for immune sensitization (Figure 1A). Cholera toxin was added as an adjuvant during sensitization to stimulate a mixed Th2/Th17 immune response, to promote cellular, humoral, and mucosal immune recall, and to prevent the development of an HDM-tolerant phenotype (Krishnamoorthy et al., 2008; Mattsson et al., 2015). There were 4 different treatment groups of mice: Naïve, Control, AAD-Vehicle (AAD no treatment), and AAD-NO_2_-OA (Figure 1B). We started by investigating the impact of NO_2_-OA on host inflammation and disease. To assess the effect of NO_2_-OA treatment on airway inflammation, we quantified the immune cells present in lung airspaces. AAD-Vehicle group had higher total BAL cell counts than Control (p< 0.0001) and Naïve (p< 0.0001) groups (Figure 1C). AAD-NO_2_-OA group had lower total BAL cells in the airways compared to AAD-Vehicle (p = 0.0118, Figure 1C). Although the BAL cell differentials were unavailable for this experiment, we had previously determined that eosinophils were the primary immune cell that increased with allergic airway disease in the model (Manni et al., 2021). In lieu of BAL cell differential counts, we measured lung mRNA expression of eosinophil granule protein proteoglycan 2, *Prg2*, and neutrophil granulocyte enzyme myeloperoxidase *Mpo* as surrogate markers for eosinophil and neutrophils, respectively. Compared to Naïve (p = 0.0421) and Control (p = 0.0331) groups, the AAD-Vehicle group had greater lung mRNA expression of *Prg2*, but no difference between the AAD-Vehicle and AAD-NO_2_-OA groups was detected (Figure 1D). *Prg2* expression was elevated for obese mice with allergic airway disease while *Mpo* expression was not detected (Figure 1D), consistent with the previously reported eosinophil and neutrophil cell counts (Manni et al., 2021). To examine cytokine signaling driving cellular inflammation in the airways, we measured lung cytokine and chemokine levels on the day of the flexiVent lung function measurement and bronchoalveolar lavage procedures by multiplex immunoassay. Compared to Naïve and Control groups, the AAD-Vehicle and AAD-NO_2_-OA groups had higher protein levels of Eotaxin-1 (CCL11) (p< 0.0001), macrophage inflammatory protein-1α (MIP-1α/CCL3, p< 0.0001), and RANTES (CCL5, p< 0.05) cytokines (Figures 1E–G). Monocyte chemoattractant protein-1 (MCP-1/CCL2) levels and lung mRNA expression of neutrophil chemokine ligand 15 (*Cxcl15*), however, were lower for AAD-Vehicle (p< 0.01) and AAD-NO_2_-OA (p< 0.01) groups compared to the Naïve and Control groups (Figure 1H; Supplement Figure 2). No differences in Eotaxin-1, MIP-1α, RANTES, and MCP-1 cytokine levels were observed between AAD-Vehicle and AAD- NO_2_-OA groups (Figures 1E–H). Obese mice with allergic airway disease had elevated eosinophil chemotactic cytokine levels and greater expression of eosinophil granule protein *Prg2* in the lung parenchyma. Obese mice with allergic airway disease treated with NO_2_-OA had lower total immune cells present in the lung airspaces.

### 3.2 NO_2_-OA alleviated elastic stiffness of lung tissue and respiratory function while expression of genes associated with mucus hypersecretion in the airways was largely driven by disease status

**FIGURE 2:**
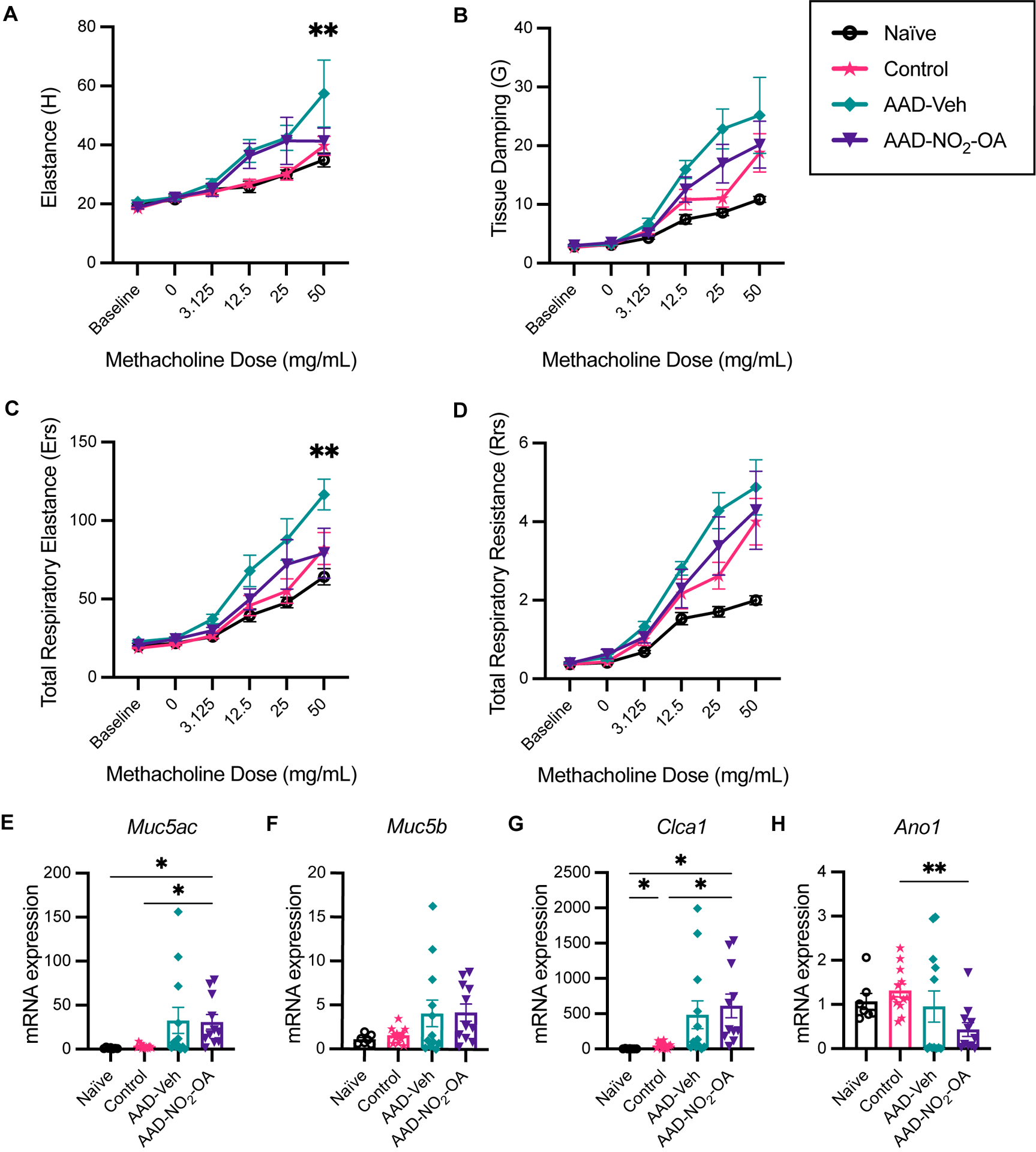
NO_2_-OA alleviated elastic stiffness of the lung tissue and respiratory system while expression of genes associated with mucus hypersecretion in the airways was largely driven by disease status. **(A)** Elastance (H), **(B)** tissue damping (G), **(C)** total respiratory elastance (Ers), and **(D)** total respiratory resistance (Rrs) were recorded at baseline and in response to increasing nebulized doses of methacholine with a flexiVent small animal ventilator. Treatment groups included obese naïve (Naïve, n = 7) marked with solid black line with circle data points, mock sensitization control (Control, n = 12) marked with solid magenta line with starred data points, AAD-Vehicle (AAD-Veh, n = 11) marked with solid teal line with diamond data points, and AAD-NO_2_-OA (n =11) marked with solid purple line with triangle data points. **(E)** *Muc5ac*, **(F)** *Muc5b*, **(G)** *Clca1*, and **(H)** *Ano1* mRNA gene expression was measured in lung tissue with RT-qPCR normalized to *Gapdh*. Treatment groups included obese naïve (Naïve, n = 7), mock sensitization control (Control, n = 12), AAD-Vehicle (AAD-Veh, n = 11), and AAD-NO_2_-OA treatment (n =11). Values are shown as mean ± SEM.Values are shown as mean ± SEM. For flexiVent lung function, statistical significance was calculated by 2-way ANOVA with Tukey’s multiple comparisons test and reported for AAD-NO_2_-OA group versus AAD-Vehicle group, **p< 0.01. Statistical significance of mRNA expression data was calculated with Welch’s ANOVA with Dunnett’s multiple comparisons test, *p< 0.05, **p< 0.01.

To examine whether NO_2_-OA treatment improved lung function, lung parameters were measured with a small animal ventilator (flexiVent, SCIREQ). Compared to AAD-Vehicle group, AAD-NO_2_-OA had lower elastance (Figure 2A, p = 0.0051) and total respiratory elastance (Figure 2C, p = 0.0024) at the 50 mg/mL methacholine dose. Tissue damping (G) at 25 mg/mL methacholine trended higher for the AAD-Vehicle group compared to the AAD-NO_2_-OA group (Figure 2B, p = 0.0629). AAD-Vehicle group had elevated elastance (Figure 2A, p< 0.0001), tissue damping (Figure 2B, p< 0.0001), total respiratory elastance (Figure 2C, p< 0.0001), and total respiratory resistance (Figure 2D, p< 0.0001) in response to 50 mg/mL methacholine bronchoprovocation compared to Naïve group. To evaluate airway mucus secretion, we measured mRNA expression of airway surface mucin 5AC (*Muc5ac*), respiratory tract mucin 5B (*Muc5b*), calcium-activated chloride channel regulator 1 (*Clca1*), and Anoctamin-1/Transmembrane member 16A protein (*Ano1*) in lung tissue. No differences were observed in mRNA expression of *Muc5ac*, *Muc5b*, *Clca1*, and *Ano1* between the AAD-Vehicle and AAD-NO_2_-OA groups (Figures 2E–H). Development of allergic airway disease with obesity worsened elastic stiffness of the lung tissue and respiratory system, increased tissue damping, and enhanced total respiratory system resistance. While NO_2_-OA treatment alleviated elastic stiffness of both the lung tissue and respiratory system, mucus hypersecretion did not improve.

### 3.3 NO_2_-OA decreased the abundance of a microbial taxon associated with poorer lung function in asthma

**FIGURE 3:**
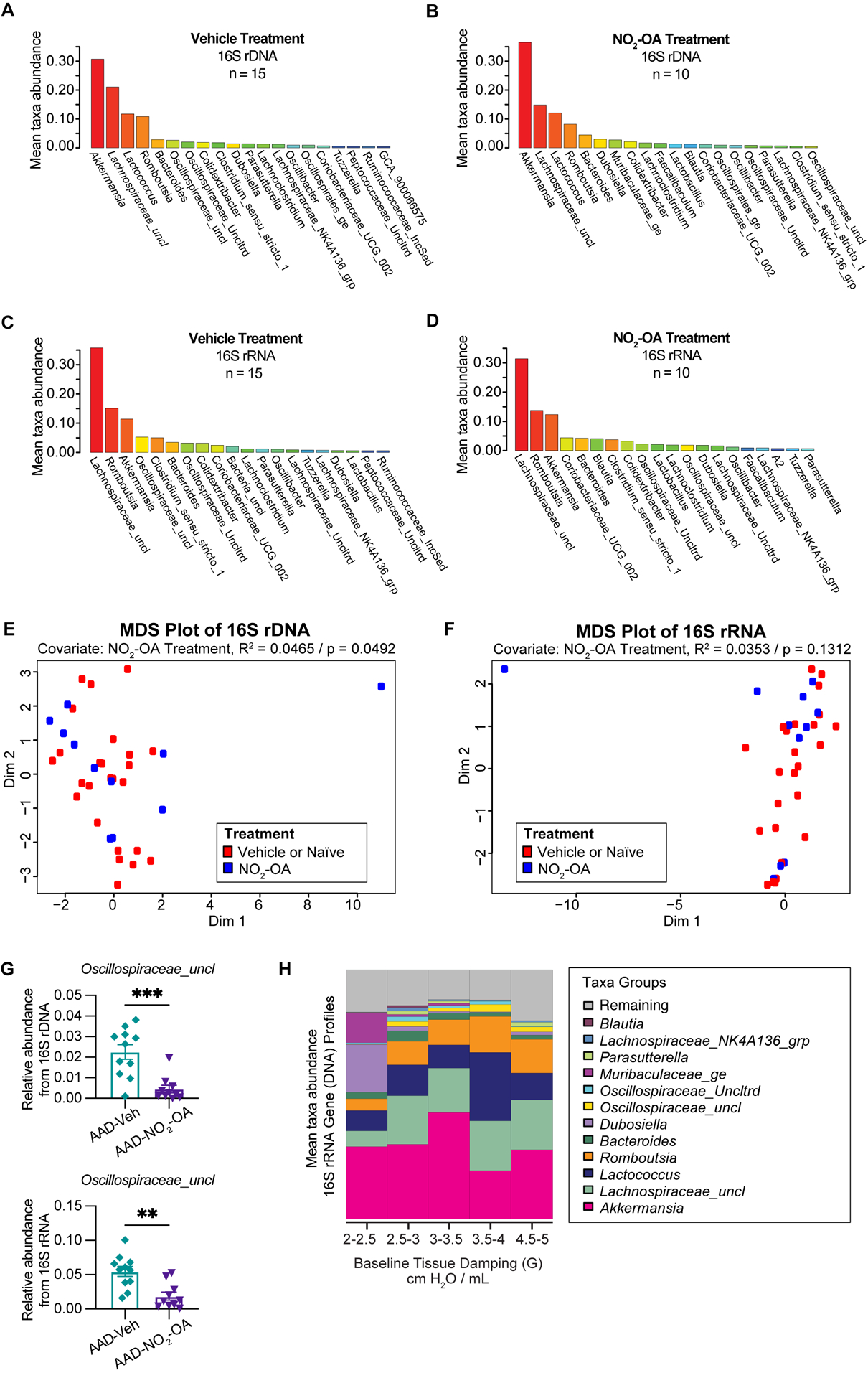
NO_2_-OA decreased the abundance of a microbial taxon associated with poorer lung function in asthma. Mean relative taxa abundances in 16S rRNA gene profiles from **(A)** AAD-Vehicle and **(B)** AAD-NO_2_-OA groups. Mean relative taxa abundances in 16S rRNA profiles from RNA (cDNA) for **(C)** AAD-Vehicle and **(D)** AAD-NO_2_-OA groups. Multidimensional scaling (MDS) plots showing inter-sample distances for **(E)** 16S rDNA and **(F)** 16S rRNA profiles paired with permutational multivariate analysis of variance (PERMANOVA) to test the association of gut microbiota composition with NO_2_-OA treatment as a covariate. **(G)** Relative abundance of *Oscillospiraceae_uncl* taxa was lower in AAD-NO_2_-OA group (n = 11) compared to AAD-Vehicle group (n = 11) in both the DNA (p = 0.0003, unpaired t-test) and RNA profiles (p = 0.0012, unpaired t-test). Values are shown as mean ± SEM. **(H)** Stacked bar chart displays mean relative abundances of major microbial taxa in DNA profiles for the following defined ranges of tissue damping, G: 2–2.5, 2.5–3.0, 3.0–3.5, 3.5–4.0, and 4.5–5.0 cmH_2_O/mL. **p<0.01 and ***p<0.001.

Next, we investigated the impact of NO_2_-OA treatment on the murine gut microbiome. To assess whether NO_2_-OA treatment modified the composition of gut microbiota, 16S profiles from stool were sequenced from both DNA and RNA (from cDNA) to determine taxa presence and activity, respectively (Figures 3A–D). To qualitatively describe the 16S profiles, the top five most abundant taxa in 16S rDNA profiles were *Akkermansia*, *Lachnospiraceae_uncl*, *Lactococcus*, *Romboutsia*, and *Bacteroides* (Figures 3A, B). The top three most abundant taxa in 16S rRNA profiles were *Lachnospiraceae_uncl*, *Romboutsia*, and *Akkermansia* (Figures 3C, D). *Akkermansia* was the most abundant taxa in the 16S rDNA profiles (Figures 3A, B), while *Lachnospiraceae_uncl* was most abundant taxa in the 16S rRNA profiles (Figures 3C, D). Multidimensional scaling (MDS) using inter-sample distances was performed for the 16S rDNA (Figure 3E) and 16S rRNA (Figure 3F) compositional data to illustrate microbial community similarity/dissimilarity with 2D spatial distances. We tested whether microbial composition in the 16S rDNA and 16S rRNA profiles differed by NO_2_-OA treatment with permutational multivariate analysis of variance (PERMANOVA). Additional covariates CT adjuvant, HDM treatment, and final weight as a measure of obesity, were also tested (Supplement Figure 3). Microbial community composition differed by NO_2_-OA treatment in 16S rDNA profiles (p = 0.0492, R^2^ = 0.0465, Figure 3E), but was not significantly different in the 16S rRNA profiles (p = 0.1312, R^2^ = 0.0353, Figure 3F). Numerous differences between AAD-NO_2_-OA and AAD-Vehicle in the less abundant major taxa were found, but the most significant difference was in the unclassified *Oscillospiraceae* taxa *(Oscillospiraceae_uncl*), where NO_2_-OA compared to Vehicle was associated with a decreased relative abundance of *Oscillospiraceae_uncl* in the gut microbiota in both the DNA (p = 0.0003) and RNA (p = 0.0012) 16S profiles (Figure 3G). We evaluated whether microbial taxa abundances were associated with lung function. The mean relative abundance of *Oscillospiraceae_uncl* increased with baseline tissue damping (G) (Figure 3H). Using multivariable regression modeling (R^2^ = 0.5525) with 16S taxa abundances as predictors and lung function parameters as responses, we found that increased abundance of *Oscillospiraceae_uncl* in the DNA profiles was associated with increased tissue damping (Table 1, p = 0.0275, β = 0.4111). Thus, NO_2_-OA treatment significantly decreased the abundance of a microbial taxon associated with increased tissue damping, a measure of increased small airway resistance and marker of poorer lung function. Relative abundance of *Lachnospiraceae_uncl* was negatively correlated with tissue damping (Table 1, p = 0.0350, β = −0.9433), but relative abundance did not change with NO_2_-OA treatment (Supplement Figure 4). NO_2_-OA treatment modified gut microbiota composition, including a microbial taxon associated with poorer lung function.

### 3.4 Meta-omics data integration and modeling reveal that gut-associated inflammation, metabolites, and functionally active gut microbiota are linked to lung function outcomes

**FIGURE 4:**
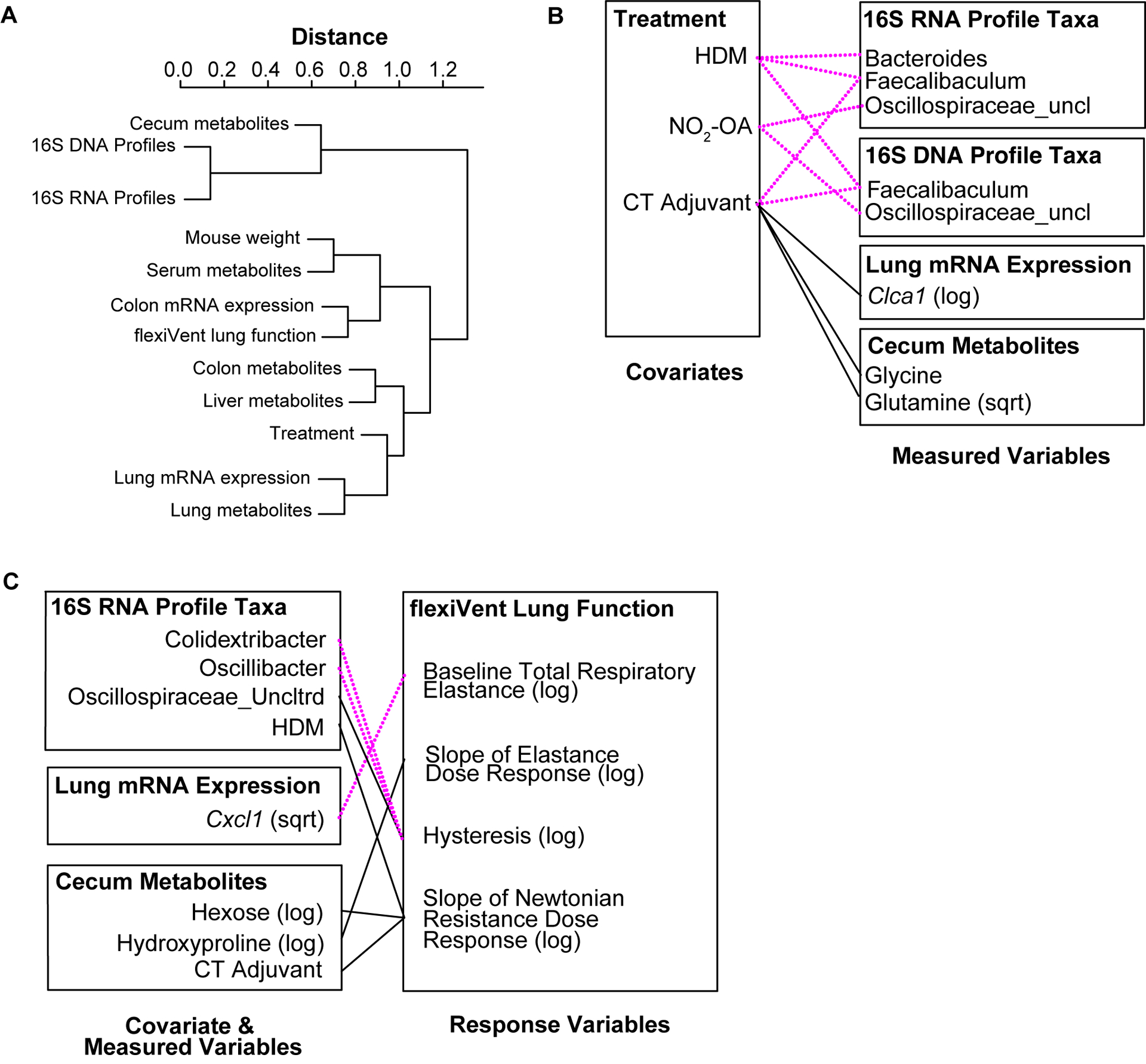
Meta-omics data integration and modeling reveal that gut-associated inflammation, metabolites, and functionally active gut microbiota are linked to lung function outcomes. **(A)** Dendrogram showing clustering and distance between meta-omics datasets. Correlations between datasets were converted to distances with the function (1 - |cor(distances)|). **(B)** Significant associations (p< 0.005) between individual variables from murine model covariates (treatments, weight) and measured data (16S, metabolomics, host gene expression). **(C)** Significant associations (p< 0.005) between individual variables from murine model covariates/measured data and response variables (flexiVent lung function). Negative associations are shown with dotted magenta line. Positive associations are marked with solid black line.

In the final phase of our study, we took a meta-omics approach to profiling the microbiome, metabolome, and host gene expression across the gut-lung axis to identify potential mechanisms of obese allergic asthma in our murine model. To understand the datasets relative to each other, we measured the inter-sample distance and clustering between groups of variables (Figure 4A), as well as correlations between inter-sample distance matrices between groups of variables (Supplement Figure 5). The similarity of variable groups based on the measurements collected for each sample and the inter-sample distance matrices calculated for each group were represented in an inter-group similarity dendrogram (Figure 4A). Combining distance-based clustering and correlation between groups of variables, we identified several key relationships. Cecum metabolites were closely clustered with 16S DNA and RNA profiles (Figure 4A). Weight (as a proxy for high fat diet) was more closely clustered with serum metabolites (Figure 4A). Treatments, including NO_2_-OA and sensitization with HDM/CT or CT only (determining allergic airway disease status), were closely clustered with lung mRNA expression and metabolites (Figure 4A). Lung function measured by flexiVent was most closely clustered with colon mRNA expression of inflammation-associated genes (Figure 4A). Close clustering may suggest signaling between compartments or similarity in the mechanisms and measured biomarkers. When we examined the individual variables links, we found numerous significant associations between covariates (weight, treatment, and sensitization by HDM/CT or CT only) and measured variables (microbial taxa, metabolites, and host gene expression) (Figure 4B). HDM (β = −7.022, p = 0.000445) and CT (β = −9.663, p = 8.246e-08) as a proxy for allergic airway disease were negatively associated with the abundance of *Faecalibaculum* in the 16S DNA profiles, as well as the 16S RNA profiles (HDM: β = −6.803, p = 6.864e-05; CT: β = −8.416, p = 3.058e-08, Figure 4B). NO_2_-OA treatment was negatively associated with the abundance of *Oscillospiraceae_uncl* in the 16S DNA (β = −2.047, p = 0.000347) and 16S RNA profiles (β = −1.738, p = 0.000451, Figure 4B). CT adjuvant was associated with increased lung mRNA expression of *Clca1* (β = 2.788, p = 0.00171), as well as increased glycine (β = 0.0545, p = 0.00103) and glutamine (β = 0.582, p = 0.000913) levels in the cecum (Figure 4B). Individual relationships between the response variables (flexiVent lung function) and covariate/measured variables were also delineated (Figure 4C). Allergic airway disease (β = 1.624, p = 0.00304) and cecum hexose levels (β = 0.945, p = 0.00474) were associated with enhanced airway hyperresponsiveness measured by Newtonian Resistance (Figure 4C). Amounts of hydroxyproline in the cecum was positively associated with lung elastance, or elastic stiffness of the lung tissue (β = 3.516, p = 0.00436, Figure 4C). In the 16S RNA profiles, *Colidextribacter* (β = −0.330, p = 0.00138) and *Oscillibacter* (β = −0.444, p = 0.000663) were negatively associated with hysteresis, while *Oscillospiraceae_Uncltrd* (β = 0.399, p = 0.000718) was positively associated (Figure 4C). Lung mRNA expression of *Cxcl1*, a neutrophil chemoattractant, was negatively associated with total respiratory elastance at baseline (β = −0.321, p = 0.00378, Figure 4C). Together, these results suggest that activity in the gut may influence lung function. Gut-associated inflammation, metabolites, and functionally active microbial taxa were associated with lung function outcomes in our murine model of obese allergic asthma.

### 3.5 Collagen and elastin constituents proline and hydroxyproline are elevated in obese allergic asthma

**FIGURE 5:**
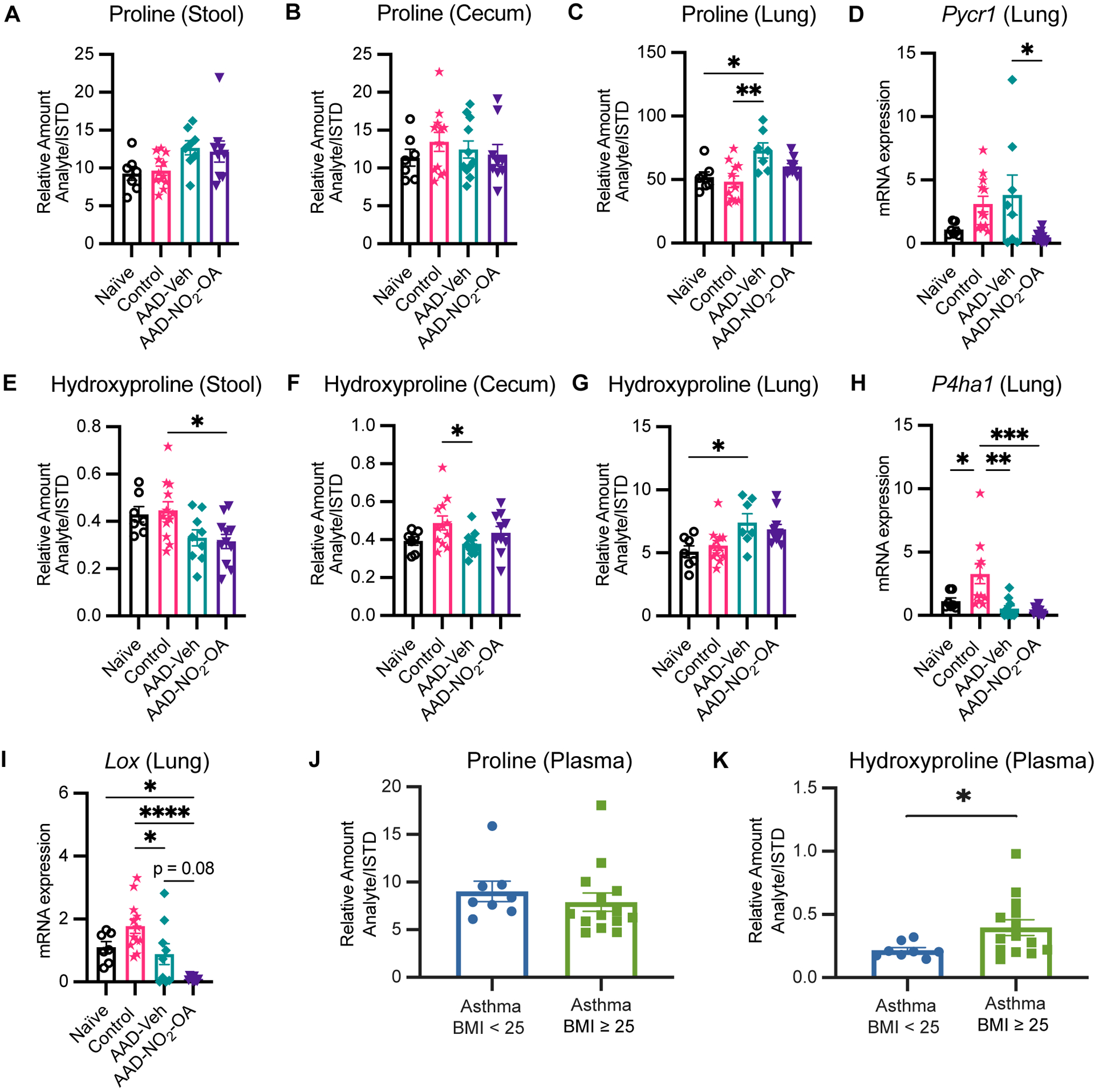
Collagen and elastin constituents proline and hydroxyproline are elevated in obese allergic asthma. Relative amount (Analyte/Internal Standard) of proline measured in murine **(A)** stool, **(B)** cecum, and **(C)** lung homogenates. **(D)** *Pycr1* mRNA gene expression was measured in lung tissue with RT-qPCR normalized to *Gapdh*. Relative amount (Analyte/Internal Standard) of hydroxyproline measured in murine **(E)** stool, **(F)** cecum, and **(G)** lung homogenates. **(H)** *P4ha1* and **(I)** *Lox* mRNA gene expression was measured in lung tissue with RT-qPCR normalized to *Gapdh*. Plasma levels of **(J)** proline and **(K)** hydroxyproline measured in adult individuals with mild-moderate asthma separated by BMI. Treatment groups in murine model included obese naïve (Naïve, n = 7), mock sensitization control (Control, n = 12), AAD-Vehicle (AAD-Veh, n = 11), and AAD-NO_2_-OA (n =11). Values are shown as mean ± SEM. Statistical significance was calculated by ordinary one-way ANOVA with Tukey’s multiple comparisons test (A-F) and unpaired t-test (J, K), *p<0.05, **p<0.01, ***p<0.001, ****p<0.0001.

To follow up on the cecum hydroxyproline and lung elastance linkage, we examined proline and hydroxyproline levels in our murine model of obese allergic asthma by disease status and found that proline and hydroxyproline in the lungs were elevated in the AAD-Vehicle group (Figures 5C, G). While no differences in cecum and stool proline were detected between treatment groups (Figures 5A, B), hydroxyproline levels in cecum and stool were lower for the AAD-Vehicle and AAD-NO_2_-OA groups, respectively, compared to the Control group (Figures 5E, F). No differences between AAD-Vehicle and AAD-NO_2_-OA groups for proline (Figures 5A, B) and hydroxyproline (Figures 5E, F) levels in the stool and cecum were detected by LC-HRMS. To identify which metabolic pathways contributed to elevated lung proline and hydroxyproline, we measured lung mRNA expression of pyrroline-5-carboxylate reductase 1 (*Pycr1*, Figure 5D) and prolyl 4-hydroxylase subunit alpha 1 (*P4ha1*, Figure 5H). PYCR1 catalyzes the final step of proline synthesis, converting pyrroline 5-carboxylate to proline, while prolyl 4-hydroxylase catalyzes the hydroxylation of proline to hydroxyproline during posttranslational modification (Gorres and Raines, 2010; Christensen et al., 2017). We observed significant differences in *Pycr1* gene expression in the lungs of mice with allergic airway disease receiving NO_2_-OA. Compared to the AAD-Vehicle group, AAD-NO_2_-OA mice had lower *Pycr1* lung mRNA expression (Figure 5D, p = 0.023). No differences in lung mRNA expression of *P4ha1* between AAD-Vehicle and AAD-NO_2_-OA groups were observed (Figure 5H). To explore whether diminished proline biosynthesis may affect downstream collagen and elastin production, we measured lung mRNA expression of lysyl oxidase (*Lox*, Figure 5I), which is responsible for cross-linking collagen and elastin precursors (Siegel et al., 1970; Kagan and Trackman, 2012). Compared to the AAD-Vehicle group, the AAD-NO_2_-OA group trended toward lower *Lox* lung mRNA expression (Figure 5H, p = 0.08). To evaluate whether these changes extended to human disease, we measured plasma amino acid levels in overweight/obese adults with mild-moderate asthma. While no differences in proline plasma levels were observed, individuals with mild-moderate asthma and a BMI ≥ 25 had higher plasma levels of hydroxyproline compared to those with asthma and a BMI< 25 (Figures 5J, K). In the murine model of obesity-associated asthma, hydroxyproline was lower in the gut, but elevated in the lungs of diseased animals. NO_2_-OA treatment inhibited proline biosynthesis, which may have downstream effects on collagen and elastin synthesis. In adults with mild-moderate asthma, plasma hydroxyproline levels were higher for overweight/obese individuals, suggesting the alterations in murine hydroxyproline levels may also be relevant to human disease.

### 3.6 Significant associations between measured variables in the treatment-measured-response model identify key interactions between allergic airway disease, NO_2_-OA, gut microbiota, and amino acid precursors to collagen and elastin

**FIGURE 6:**
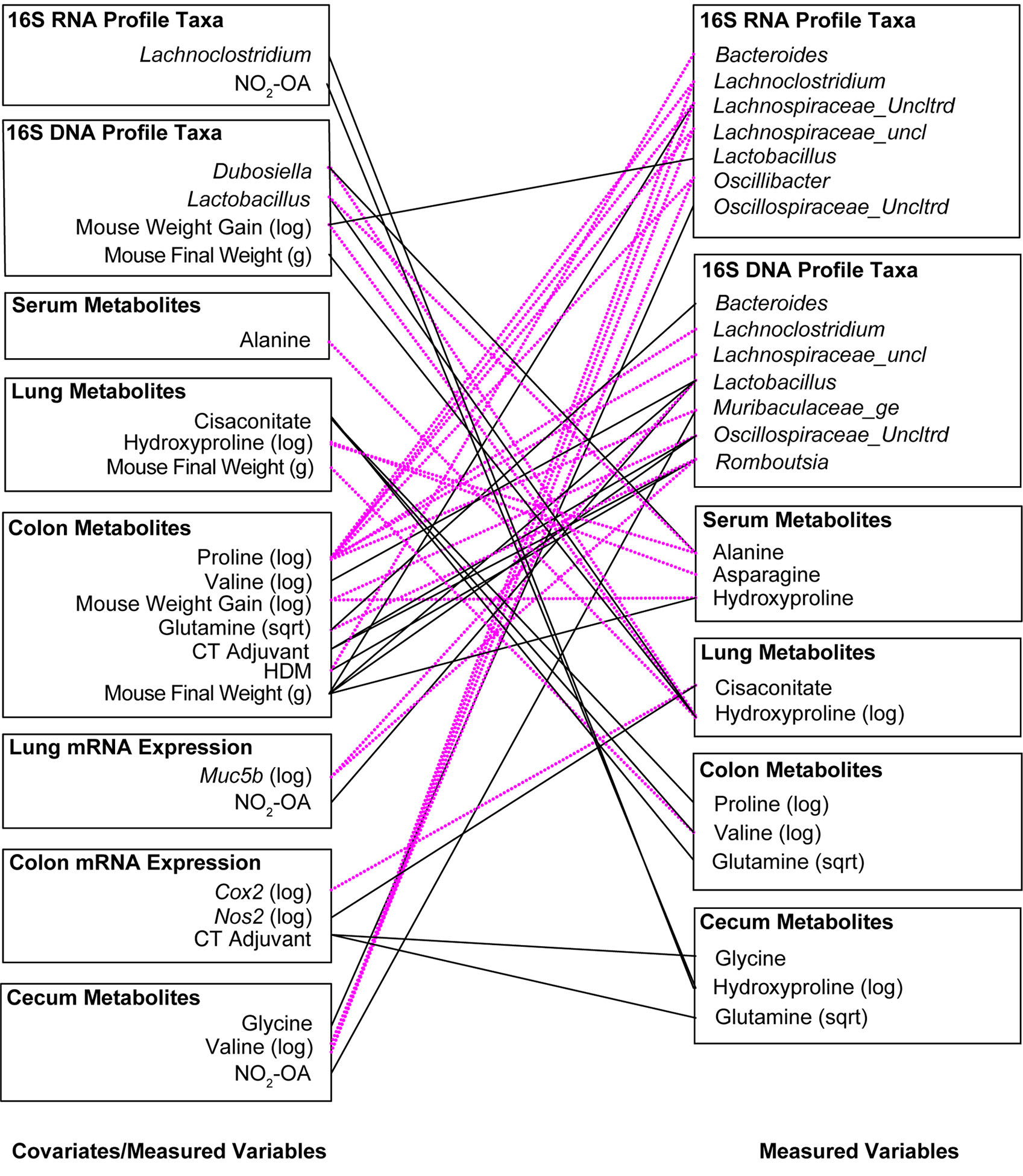
Significant associations (p< 0.005) between measured variables in the Treatment-Measured-Response Model identify key interactions between allergic airway disease, NO_2_-OA, gut microbiota, and amino acid precursors to collagen and elastin. Negative associations are shown with dotted magenta line. Positive associations are marked with solid black line.

To examine latent (hidden) relationships among the measured variables (16S, metabolomics, host gene expression) that might explain additional host-microbial interactions, significant associations (p< 0.005) between measured variables were identified in the Treatment-Measured-Response model (Supplement Table 1) and summarized visually (Figure 6). Several microbial taxa were linked to allergic airway disease status. Receiving CT adjuvant during sensitization was associated with greater *Romboutsia* (β = 3.189, p = 0.000176) and *Oscillospiraceae_Uncltrd* (β = 3.242, p = 0.00303) in 16S DNA taxa profiles (Figure 6; Supplement Table 1). Sensitization with HDM (allergic airway disease) was connected to lower *Bacteriodes* in16S RNA, (β = −2.661, p = 0.000396), but greater *Romboutsia* (β = 3.879, p = 0.000150) in 16S DNA taxa profiles (Figure 6, Supplement Table 1). Development of allergic airway disease was associated with lower *Bacteriodes*, but greater abundance of *Romboutsia*, in the gut microbiome (Figure 6; Supplement Table 1). We observed significant interactions between amino acids levels in the gut and abundance of gut microbiota (Figure 6; Supplement Table 1). Particularly of interest were cecum hydroxyproline and stool proline levels given associations observed with lung elastance. Stool proline levels were negatively associated with multiple gut microbiota: *Lachnospiraceae_uncl* (16S DNA, β = −3.661, p = 0.000253 and 16S RNA, β =−3.381, p = 0.00206) *Muribaculaceae_ge* (16S DNA, β = −4.420, p = 0.00318), *Lachnoclostridium* (16S DNA, β = −3.749, p = 0.00135 and 16S RNA, β = −4.190, p = 0.000288), *Oscillospiraceae_Uncltrd* (16S RNA, β = −3.441, p = 0.00109), and *Oscillibacter* (16S RNA, β = −3.767, p = 0.000085) (Figure 6; Supplement Table 1). NO_2_-OA treatment, which alleviated lung elastic stiffness and total respiratory elastance, was linked to greater *Muribaculaceae_ge* (β = 2.345, p = 0.00423) and *Lactobacillus* (β = 3.592, p = 0.00115) in 16S DNA taxa profiles (Figure 6; Supplement Table 1), suggesting that NO_2_-OA may be associated with microbial proline metabolism in the gut as well. Using Treatment-Measured-Response modeling, a previously hidden network of host-microbe interactions between gut levels of amino acid metabolites, gut microbiota, NO_2_-OA treatment, and lung elastic stiffness emerged.

## 4 Discussion

**FIGURE 7:**
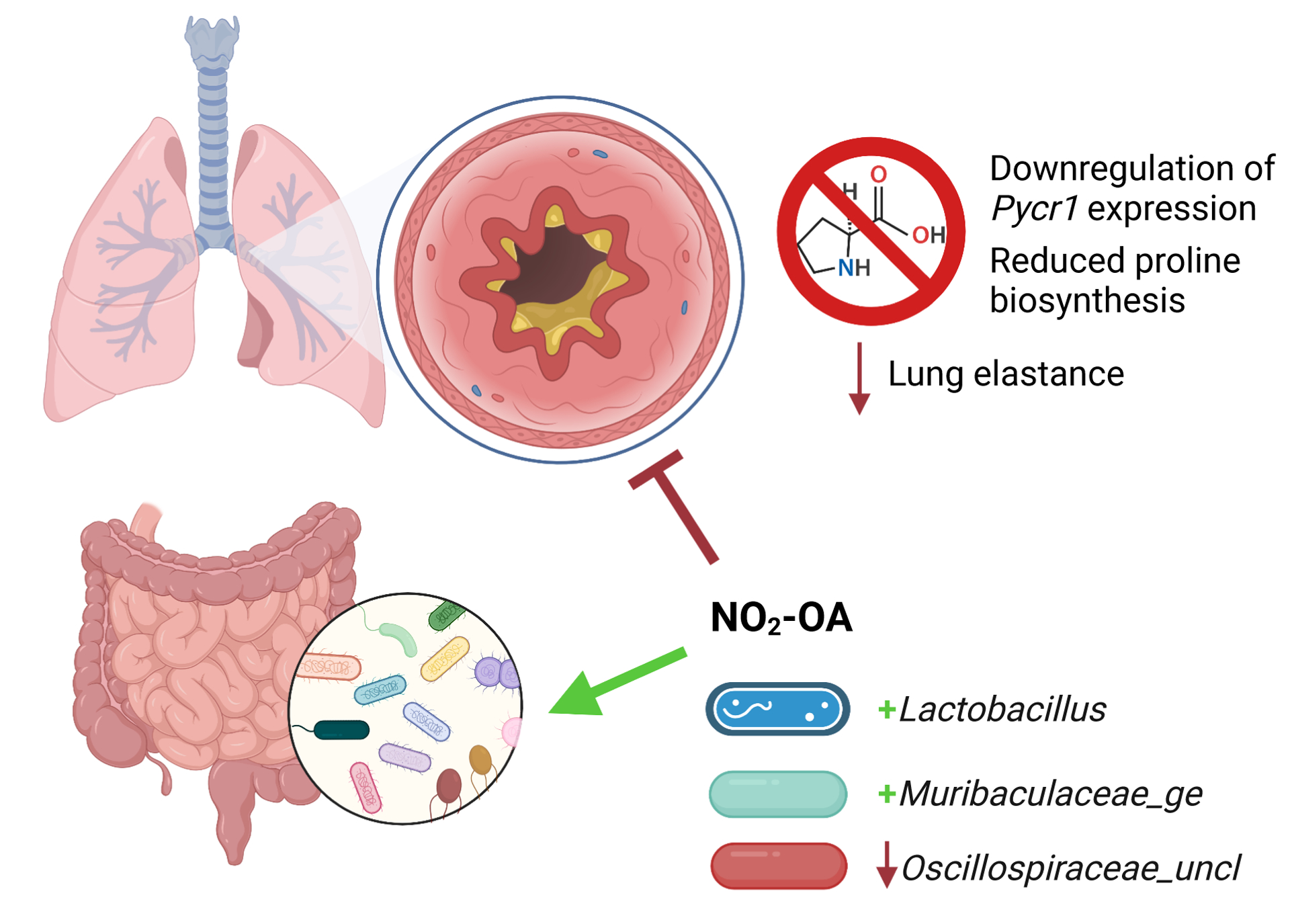
Nitro-oleic acid (NO_2_-OA) targeted both the gut microbiota and host lungs in a murine model of obese allergic asthma. NO_2_-OA positively impacted the gut microbiota and was associated with increased abundance of anti-inflammatory taxa *Lactobacillus* and *Muribaculaceae_ge*. NO_2_-OA decreased the abundance of *Oscillospi*raceae_uncl, which was associated increased tissue damping and poorer lung function. NO_2_-OA downregulated Pycr1 expression and proline biosynthesis in the lung parenchyma and lowered lung elastance, leading to improved lung function.

We tested NO_2_-OA as a treatment for obese allergic asthma and evaluated its impact on the gut microbiome-metabolome of a murine model. NO_2_-OA alleviated airway inflammation as measured by total immune cell numbers and lung elastance, but did not improve mucus hypersecretion compared with untreated control obese mice with allergic airway disease. NO_2_-OA was linked to several key modifications of the gut microbiota. NO_2_-OA reduced levels of *Oscillospiraceae_uncl*, a bacterial taxon that was associated with greater tissue damping (G), a measure of small airway resistance and poor lung function. NO_2_-OA treatment was also linked to greater abundance of beneficial microbial taxa, *Lactobacillus* and *Muribaculaceae_ge*, which were negatively associated with stool proline in our model. These associations identified in our study model suggest that NO_2_-OA treatment may benefit both the host and the gut microbiota in obese allergic asthma and warrant further investigation (Figure 7).

This study presents preclinical evidence for the ability of NO_2_-OA to modulate lung function in obesity-associated asthma, with prior studies reinforcing that the translation of NO_2_-OA for obese allergic asthma may also prove effective in treating human disease. For example, clinical elevation of lung elastance in obese adults with asthma has been reported (Bates et al., 2021), indicating that NO_2_-OA targets lung mechanics relevant to human disease. Consuming a Mediterranean diet rich in olive oil, a dietary source of fatty-acid nitroalkenes, has been associated with improved asthma control and lower risk of developing asthma in adults and children (Chatzi et al., 2007; Garcia-Marcos et al., 2007; Barros et al., 2008; Fazzari et al., 2014; Cazzoletti et al., 2019). Nitration of extra virgin olive oil in the acidic environment of the stomach has been shown to produce endogenous nitro-oleic acid (Fazzari et al., 2014). A clinical trial (NCT03762395) of the specific positional isomer 10-NO_2_-OA (CP-6) is currently underway in adults with obesity-associated asthma. The present study reinforces the rationale for this Phase 2 trial using a relevant model system that expands upon the well-documented, systemic anti-inflammatory effects of NO_2_-OA treatment in the host (Cui et al., 2006; Delmastro-Greenwood et al., 2014; Ambrozova et al., 2016; Schopfer et al., 2018).

Given NO_2_-OA is predominantly absorbed in the gut and regulates microbial-derived metabolites such as bile acids, prior studies have postulated that NO_2_-OA modifies the gut microbiome and host-microbial metabolism (Fazzari et al., 2017; Manni et al., 2021), which is affirmed by this study. Moreover, the gut microbiota affected by NO_2_-OA include microbiota relevant to asthma in murine models and humans. Supplementation with *Lactobacillus*, which was positively associated with NO_2_-OA treatment in our study, suppresses lung inflammation in other murine models of allergic airway disease (Wu et al., 2016). Oral administration of *Lactobacillus* reduces airway inflammation and symptoms in school-age children with asthma and restores anti-inflammatory fatty acid metabolites in human infants at high risk for developing asthma (Chen et al., 2010; Miraglia Del Giudice et al., 2012; Durack et al., 2018). Studies to date have focused on *Lactobacillus* supplementation as a preventative and therapeutic strategy for childhood asthma (Chen et al., 2010; Miraglia Del Giudice et al., 2012; Durack et al., 2018). Given the positive association between *Lactobacillus* and NO_2_-OA in this study and other groups’ findings that *Lactobacillus* supplementation attenuates airway inflammation, the therapeutic potential of *Lactobacillus* supplementation paired with a fatty acid nitroalkene prebiotic, such as NO_2_-OA, conjugated linoleic acid, or olive oil, is a possible future direction for consideration in obese allergic asthma.

We also sought to identify potential molecular mechanisms of obese allergic asthma using a meta-omics profiling approach in our murine model. This meta-omics profiling and Treatment-Measured-Response data integration model revealed a network of connections between proline and hydroxyproline levels in the gut, gut microbiota, and greater lung elastance. *Lachnospiraceae*, one of the 16S taxa that was associated with lower stool proline, expresses proline reductase *PrdA* and has been reported to compete with other microbiota for proline and other amino acids (Mefferd et al., 2020). *Muribaculaceae*, which was also linked to lower stool proline in our model, is associated with anti-obesity effects and produces butyrate, a short chain fatty acid that attenuates lung inflammation in murine models of allergic airway disease (Cait et al., 2017; Xu et al., 2020; Ye et al., 2021; Lv et al., 2022). However, we did not observe changes in short chain fatty acids measured from cecal tissue in this model (Supplement Figure 6). Both lung elastance and *Muribaculaceae_ge* were associated with NO_2_-OA treatment, as NO_2_-OA lowered lung elastance and was linked to greater abundance of *Muribaculaceae_ge*. Further investigation using targeted metabolomics analysis revealed that obese mice with allergic airway disease had higher levels of proline and hydroxyproline in the lungs and that asthma patients with BMI ≥ 25 had higher plasma hydroxyproline levels. Obese mice with allergic airway disease treated with NO_2_-OA had diminished proline biosynthesis in the lungs. Proline is abundant in structural proteins elastin and collagen, which are present in the airways and lung parenchyma (Gorres and Raines, 2010). A fraction of the proline residues in elastin and collagen undergo posttranslational modification to hydroxyproline by prolyl 4-hydroxylase (Gorres and Raines, 2010). While elastin provides strength and flexibility to the lung tissue, increased submucosal elastin and airway thickening were observed in bronchial biopsies from adults with severe asthma and an average BMI > 30 (Wilson et al., 2021). Collagen deposition can also increase elastic stiffness of airways and lung tissue (Li and Wu, 2018; Hough et al., 2020; Karna et al., 2020). NO_2_-OA treatment also promotes collagen degradation by tissue resident macrophages in murine models of lung fibrosis (Koudelka et al., 2022). We speculate that a possible mechanism by which NO_2_-OA attenuates lung elastance is by the modulation of gut proline metabolism by microbiota and downregulation of lung proline biosynthesis by pyrroline-5-carboxylate reductase 1 (PYCR1), leading to reduced deposition of elastin and collagen in the lungs.

Although our findings provide evidence for the multi-target immunomodulator NO_2_-OA modifying lung function, inflammatory responses, and the gut microbiome in obese allergic asthma, the link between NO_2_-OA, allergic airway disease, and gut microbiome is complex. Future studies using germ free mice will help to determine the extent to which the therapeutic benefits of NO_2_-OA treatment are mediated by the host versus the gut microbiota. In our present work, meta-omics profiling and analysis was used to pinpoint connections between lung function and specific gut microbiota. These associations uncovered by our meta-omics profiling and data integration model reveal potential disease mechanisms through the networks of connections. Our objective in developing a meta-omics profiling and data integration model was to elucidate potential mechanisms of obese allergic asthma involving the gut microbiome for future studies with mechanism-focused hypothesis testing. While a murine model of obese allergic asthma allowed for extensive meta-omics profiling of host tissues along the gut-lung axis, some of the identified relationships in our model are murine-specific. For instance, NO_2_-OA treatment was positively associated with beneficial microbial taxa *Muribaculaceae_ge*, which is found in the intestinal microbiome of rodents (Lagkouvardos et al., 2019). Future human studies will help to identify comparable, human-specific gut microbiota that respond to NO_2_-OA treatment. The clinical study design of the ongoing Phase 2 trial of 10-NO_2_-OA in adults with obesity-associated asthma includes pre- and post-10-NO_2_-OA administration fecal material collections for future profiling of human-specific gut microbiota responses. Although some murine-specific associations were uncovered in the Treatment-Measured-Response model, numerous relationships that were identified were relevant to human disease. *Faecalibaculum*, which was negatively associated with allergic airway disease in our murine model, is also negatively correlated with symptoms in chronic rhinosinusitis patients, suggesting *Faecalibaculum* supplementation may benefit allergic respiratory diseases (Gan et al., 2021). Despite its limitations, a murine model paired with meta-omics analysis illuminates additional potential targets and treatment options for obese allergic asthma.

In summary, we conclude that oral NO_2_-OA administration may represent a beneficial treatment for obesity-associated asthma as evidenced by an ability to modulate immune cell infiltration or accumulation in the airways, improve lung elastance, and positively modulate the gut microbiota in our murine model. We introduced a meta-omics profiling approach of 16S rRNA and rRNA gene sequence, metabolomics, and host gene expression data with a Treatment-Measured-Response variable model for data integration and identification of hidden relationships within the data, providing a strategy to address an important technical knowledge gap for the field (Kozik et al., 2022). This novel approach enabled us to identify proline metabolism and the inhibition of the synthesis of proline-rich, structural proteins elastin and collagen as a potential mechanism accounting for the ability of NO_2_-OA to relieve lung elastance. While other studies have proposed that enhanced lung elastance stems from increased adiposity and chest wall weight in obesity (Bates et al., 2021), the present results suggest that changes to structural proteins in the lung airways and parenchyma may also contribute to heightened lung elastance and serve as a potential therapeutic target for obese allergic asthma. Targeted inhibition of proline biosynthesis in the lung extracellular matrix may mitigate aberrant elastin accumulation observed in fatal asthma and airway remodeling due to increased collagen deposition in uncontrolled, corticosteroid-resistant asthma (Araujo et al., 2008; Burgstaller et al., 2017). Probiotic supplementation with gut microbiota that attenuate lung inflammation, such as *Lactobacillus*, may also augment the therapeutic benefits of NO_2_-OA in obese allergic asthma. Leveraging synergistic interactions between future therapies and the gut microbiota is an important consideration for treating obese allergic asthma, a disease process involving both the host and microbiota.

## Supplementary Material

Supplementary Material

## Figures and Tables

**TABLE 1:** Gut microbiota abundance predicts tissue damping (G) at baseline.

Predictor variables (x_n_)	Coefficient (ß)	SE	t-value	p-value
*Akkermansia*	−0.0437	0.1263	−0.3458	0.7366
*Lachnospiraceae_uncl*	−0.9433	0.3871	−2.4371	0.0350*
*Lactococcus*	0.2902	0.2813	1.0318	0.3265
*Romboutsia*	0.2511	0.1320	1.9021	0.0863
*Bacteroides*	0.3176	0.2730	1.1633	0.2717
*Dubosiella*	0.0250	0.1055	0.2366	0.8177
*Colidextribacter*	−0.3699	0.3380	−1.0946	0.2994
*Oscillospiraceae_uncl*	0.4111	0.1594	2.5791	0.0275*
*Oscillospiraceae_Uncltrd*	0.2683	0.2026	1.3242	0.2149
*Lachnoclostridium*	0.3364	0.2051	1.6406	0.1320

Response variable (Y): Tissue damping (G) at baseline.

* p<0.05.

## Data Availability

The data presented in the study are deposited in the NCBI repository https://www.ncbi.nlm.nih.gov/, accession number PRJNA940462.

## References

[R1] Ambrozova G, Martiskova H, Koudelka A, Ravekes T, Rudolph TK, Klinke A, (2016). Nitro-oleic acid modulates classical and regulatory activation of macrophages and their involvement in pro-fibrotic responses. Free Radic. Biol. Med 90, 252–260. doi: 10.1016/J.FREERADBIOMED.2015.11.026 26620549 PMC4748956

[R2] Araujo BB, Dolhnikoff M, Silva LFF, Elliot J, Lindeman JHN, Ferreira DS, (2008). Extracellular matrix components and regulators in the airway smooth muscle in asthma. Eur. Respir. J 32, 61–69. doi: 10.1183/09031936.00147807 18321931

[R3] Barros R, Moreira A, Fonseca J, Ferraz De Oliveira J, Delgado L, Castel-Branco MG, (2008). Adherence to the Mediterranean diet and fresh fruit intake are associated with improved asthma control. Allergy 63, 917–923. doi: 10.1111/J.1398-9995.2008.01665.X 18588559

[R4] Bates JHT, Peters U, Daphtary N, MacLean ES, Hodgdon K, Kaminsky DA, (2021). Altered airway mechanics in the context of obesity and asthma. J. Appl. Physiol 130, 36–47. doi: 10.1152/JAPPLPHYSIOL.00666.2020/ASSET/IMAGES/LARGE/AJ-JAPP200089F008.JPEG 33119471 PMC7944930

[R5] Borniquel S, Jansson EÅ, Cole MP, Freeman BA, and Lundberg JO (2010). Nitrated oleic acid up-regulates PPARg and attenuates experimental inflammatory bowel disease. Free Radic. Biol. Med 48, 499–505. doi: 10.1016/J.FREERADBIOMED.2009.11.014 19932165 PMC3290869

[R6] Burgstaller G, Oehrle B, Gerckens M, White ES, Schiller HB, and Eickelberg O (2017). The instructive extracellular matrix of the lung: basic composition and alterations in chronic lung disease. Eur. Respir. J 50, 1601805. doi: 10.1183/13993003.01805-2016 28679607

[R7] Cait A, Hughes MR, Antignano F, Cait J, Dimitriu PA, Maas KR, (2017). Microbiome-driven allergic lung inflammation is ameliorated by short-chain fatty acids. Mucosal Immunol. 11, 785–795. doi: 10.1038/mi.2017.75 29067994

[R8] Caporaso JG, Lauber CL, Walters WA, Berg-Lyons D, Huntley J, Fierer N, (2012). Ultra-high-throughput microbial community analysis on the illumina HiSeq and MiSeq platforms. ISME J. 6, 1621–1624. doi: 10.1038/ismej.2012.8 22402401 PMC3400413

[R9] Cazzoletti L, Zanolin ME, Spelta F, Bono R, Chamitava L, Cerveri I, (2019). Dietary fats, olive oil and respiratory diseases in Italian adults: a population-based study. Clin. Exp. Allergy 49, 799–807. doi: 10.1111/CEA.13352 30689281

[R10] Chatzi L, Apostolaki G, Bibakis I, Skypala I, Bibaki-Liakou V, Tzanakis N, (2007). Protective effect of fruits, vegetables and the Mediterranean diet on asthma and allergies among children in Crete. Thorax 62, 677–683. doi: 10.1136/THX.2006.069419 17412780 PMC2117278

[R11] Chen YS, Lin YL, Jan RL, Chen HH, and Wang JY (2010). Randomized placebo-controlled trial of lactobacillus on asthmatic children with allergic rhinitis. Pediatr. Pulmonol 45, 1111–1120. doi: 10.1002/PPUL.21296 20658483

[R12] Cho Y, Abu-Ali G, Tashiro H, Kasahara DI, Brown TA, Brand JD, (2018). The microbiome regulates pulmonary responses to ozone in mice. Am. J. Respir. Cell Mol. Biol 59, 346–354. doi: 10.1165/rcmb.2017-0404OC 29529379 PMC6189641

[R13] Christensen EM, Patel SM, Korasick DA, Campbell AC, Krause KL, Becker DF, (2017). Resolving the cofactor-binding site in the proline biosynthetic enzyme human pyrroline-5-carboxylate reductase 1. J. Biol. Chem 292, 7233–7243. doi: 10.1074/jbc.M117.780288 28258219 PMC5409489

[R14] Cole JR, Wang Q, Cardenas E, Fish J, Chai B, Farris RJ, (2009). The ribosomal database project: improved alignments and new tools for rRNA analysis. Nucleic Acids Res. 37, D141–D145. doi: 10.1093/NAR/GKN879 19004872 PMC2686447

[R15] Cui T, Schopfer FJ, Zhang J, Chen K, Ichikawa T, Baker PRS, (2006). Nitrated fatty acids: endogenous anti-inflammatory signaling mediators. J. Biol. Chem 281, 35686–35698. doi: 10.1074/JBC.M603357200 16887803 PMC2169500

[R16] Delmastro-Greenwood M, Freeman BA, and Wendell SG (2014). Redox-dependent anti-inflammatory signaling actions of unsaturated fatty acids. Annu. Rev. Physiol 76, 79–105. doi: 10.1146/annurev-physiol-021113-170341 24161076 PMC4030715

[R17] Dixon AE, Pratley RE, Forgione PM, Kaminsky DA, Whittaker-Leclair LA, Griffes LA, (2011). Effects of obesity and bariatric surgery on airway hyperresponsiveness, asthma control, and inflammation. J. Allergy Clin. Immunol 128, 508–515.e2. doi: 10.1016/j.jaci.2011.06.009 21782230 PMC3164923

[R18] Durack J, Kimes NE, Lin DL, Rauch M, McKean M, McCauley K, (2018). Delayed gut microbiota development in high-risk for asthma infants is temporarily modifiable by lactobacillus supplementation. Nat. Commun 9, 1–9. doi: 10.1038/s41467-018-03157-4 29453431 PMC5816017

[R19] Fajt ML, Gelhaus SL, Freeman B, Uvalle CE, Trudeau JB, Holguin F, (2013). Prostaglandin D2 pathway upregulation: relation to asthma severity, control, and TH2 inflammation. J. Allergy Clin. Immunol 131, 1504–1512.e12. doi: 10.1016/j.jaci.2013.01.035 23506843 PMC3889167

[R20] Fazzari M, Khoo NKH, Woodcock SR, Jorkasky DK, Li L, Schopfer FJ, (2017). Nitro-fatty acid pharmacokinetics in the adipose tissue compartment. J. Lipid Res 58, 375–385. doi: 10.1194/JLR.M072058 27913584 PMC5282953

[R21] Fazzari M, Trostchansky A, Schopfer FJ, Salvatore SR, Sánchez-Calvo B, Vitturi D, (2014). Olives and olive oil are sources of electrophilic fatty acid nitroalkenes. PloS One 9, e84884. doi: 10.1371/JOURNAL.PONE.0084884 24454759 PMC3891761

[R22] Gan W, Zhang H, Yang F, Liu S, Liu F, and Meng J (2021). The influence of nasal microbiome diversity and inflammatory patterns on the prognosis of nasal polyps. Sci. Rep 11, 1–12. doi: 10.1038/s41598-021-85292-5 33737534 PMC7973562

[R23] Garcia-Marcos L, Canflanca IM, Garrido JB, Varela ALS, Garcia-Hernandez G, Grima FG, (2007). Relationship of asthma and rhinoconjunctivitis with obesity, exercise and Mediterranean diet in Spanish schoolchildren. Thorax 62, 503–508. doi: 10.1136/THX.2006.060020 17251311 PMC2117202

[R24] Gorres KL, and Raines RT (2010). Prolyl 4-hydroxylase. Crit. Rev. Biochem. Mol. Biol 45, 106. doi: 10.3109/10409231003627991 20199358 PMC2841224

[R25] Greiner B, and Hartwell M (2022). Prevalence and associations between metabolically unhealthy obesity and asthma exacerbations and emergency department usage. Ann. Allergy Asthma Immunol 129 (5), 580–584.e2. doi: 10.1016/J.ANAI.2022.07.005 35843518

[R26] Hough KP, Curtiss ML, Blain TJ, Liu RM, Trevor J, Deshane JS, (2020). Airway remodeling in asthma. Front. Med 7. doi: 10.3389/FMED.2020.00191/BIBTEX PMC7253669 32509793

[R27] Kagan HM, and Trackman PC (2012). Properties and function of lysyl oxidase. Am. J. Respir. Cell Mol. Biol 5, 206–210. doi: 10.1165/AJRCMB/5.3.206 1680355

[R28] Karna E, Szoka L, Huynh TYL, and Palka JA (2020). Proline-dependent regulation of collagen metabolism. Cell. Mol. Life Sci 77, 1911–1918. doi: 10.1007/S00018-019-03363-3/FIGURES/2 31740988 PMC7228914

[R29] Kelley EE, Baust J, Bonacci G, Golin-Bisello F, Devlin JE, St. Croix CM, (2014). Fatty acid nitroalkenes ameliorate glucose intolerance and pulmonary hypertension in high-fat diet-induced obesity. Cardiovasc. Res 101, 352–363. doi: 10.1093/CVR/CVT341 24385344 PMC3928004

[R30] Khoo NKH, Fazzari M, Chartoumpekis DV, Li L, Guimaraes DA, Arteel GE, (2019). Electrophilic nitro-oleic acid reverses obesity-induced hepatic steatosis. Redox Biol. 22 doi: 10.1016/J.REDOX.2019.101132 PMC6375063 30769284

[R31] Koudelka A, Cechova V, Rojas M, Mitash N, Bondonese A, St. Croix C, (2022). Fatty acid nitroalkene reversal of established lung fibrosis. Redox Biol. 50, 102226. doi: 10.1016/J.REDOX.2021.102226 35150970 PMC8844680

[R32] Kozik AJ, Holguin F, Segal LN, Chatila TA, Dixon AE, Gern JE, (2022). Microbiome, metabolism, and immunoregulation of asthma: an American thoracic society and national institute of allergy and infectious diseases workshop report. Am. J. Respir. Cell Mol. Biol 67, 155–163. doi: 10.1165/RCMB.2022-0216ST 35914321 PMC9348558

[R33] Krishnamoorthy N, Oriss TB, Paglia M, Fei M, Yarlagadda M, Vanhaesebroeck B, (2008). Activation of c-kit in dendritic cells regulates T helper cell differentiation and allergic asthma. Nat. Med 14, 565–573. doi: 10.1038/nm1766 18454155 PMC3664066

[R34] Lagkouvardos I, Lesker TR, Hitch TCA, Gálvez EJC, Smit N, Neuhaus K, (2019). Sequence and cultivation study of muribaculaceae reveals novel species, host preference, and functional potential of this yet undescribed family. Microbiome 7, 1–15. doi: 10.1186/S40168-019-0637-2/FIGURES/4 30782206 PMC6381624

[R35] Ley RE, Turnbaugh PJ, Klein S, and Gordon JI (2006). Microbial ecology: human gut microbes associated with obesity. Nature 444, 1022–1023. doi: 10.1038/4441022A 17183309

[R36] Li P, and Wu G (2018). Roles of dietary glycine, proline, and hydroxyproline in collagen synthesis and animal growth. Amino Acids 50, 29–38. doi: 10.1007/S00726-017-2490-6/TABLES/7 28929384

[R37] Litonjua AA, Sparrow D, Celedon JC, DeMolles D, and Weiss ST (2002). Association of body mass index with the development of methacholine airway hyperresponsiveness in men: the normative aging study. Thorax 57, 581–585. doi: 10.1136/THORAX.57.7.581 12096199 PMC1746377

[R38] Livak KJ, and Schmittgen TD (2001). Analysis of relative gene expression data using real-time quantitative PCR and the 2–ΔΔCT method. Methods 25, 402–408. doi: 10.1006/METH.2001.1262 11846609

[R39] Lv Y, Zhang Y, Feng J, Zhao T, Zhao J, Ge Y, (2022). (20R)-panaxadiol as a natural active component with anti-obesity effects on ob/ob mice via modulating the gut microbiota. Mol 27, 2502. doi: 10.3390/MOLECULES27082502 PMC9032863 35458705

[R40] Manni ML, Heinrich VA, Buchan GJ, O’Brien JP, Uvalle C, Cechova V, (2021). Nitroalkene fatty acids modulate bile acid metabolism and lung function in obese asthma. Sci. Rep 11, 1–12. doi: 10.1038/s41598-021-96471-9 34493738 PMC8423735

[R41] Manni ML, Mandalapu S, McHugh KJ, Elloso MM, Dudas PL, and Alcorn JF (2016). Molecular mechanisms of airway hyperresponsiveness in a murine model of steroid-resistant airway inflammation. J. Immunol 196, 963–977. doi: 10.4049/JIMMUNOL.1501531 26729801 PMC4724491

[R42] Manni ML, Trudeau JB, Scheller EV, Mandalapu S, Elloso MM, Kolls JK, (2014). The complex relationship between inflammation and lung function in severe asthma. Mucosal Immunol. 7, 1186–1198. doi: 10.1038/mi.2014.8 24549277 PMC4138304

[R43] Mantel N (1967). The detection of disease clustering and a generalized regression approach. Cancer Res. 27, 209–220. 6018555

[R44] Mattsson J, Schön K, Ekman L, Fahlén-Yrlid L, Yrlid U, and Lycke NY (2015). Cholera toxin adjuvant promotes a balanced Th1/Th2/Th17 response independently of IL-12 and IL-17 by acting on gsα in CD11b+ DCs. Mucosal Immunol. 8, 815–827. doi: 10.1038/MI.2014.111 25425266

[R45] Mefferd CC, Bhute SS, Phan JR, Villarama JV, Do DM, Alarcia S, (2020). A high-Fat/High-Protein, Atkins-type diet exacerbates clostridioides (Clostridium) difficile infection in mice, whereas a high-carbohydrate diet protects. mSystems 5, (1), e00765–19. doi: 10.1128/MSYSTEMS.00765-19/SUPPL_FILE/MSYSTEMS.00765-19-ST004.EPS PMC7018531 32047064

[R46] Miraglia Del Giudice M, Maiello N, Decimo F, Fusco N, D’Agostino B, Sullo N, (2012)Airways allergic inflammation and l. reuterii treatment in asthmatic children (Accessed August 22, 2022). 22691248

[R47] Moore WC, Evans MD, Bleecker ER, Busse WW, Calhoun WJ, Castro M, (2011). Safety of investigative bronchoscopy in the severe asthma research program. J. Allergy Clin. Immunol 128, 328–336.e3. doi: 10.1016/j.jaci.2011.02.042 21496892 PMC3149754

[R48] Mosen DM, Schatz M, Magid DJ, and Camargo CA (2008). The relationship between obesity and asthma severity and control in adults. J. Allergy Clin. Immunol 122, 507–511.e6. doi: 10.1016/J.JACI.2008.06.024 18774387

[R49] Quast C, Pruesse E, Yilmaz P, Gerken J, Schweer T, Yarza P, (2013). The SILVA ribosomal RNA gene database project: improved data processing and web-based tools. Nucleic Acids Res. 41, D590. doi: 10.1093/NAR/GKS1219 23193283 PMC3531112

[R50] Schatz M, Hsu JWY, Zeiger RS, Chen W, Dorenbaum A, Chipps BE, (2014). Phenotypes determined by cluster analysis in severe or difficult-to-treat asthma. J. Allergy Clin. Immunol 133, 1549–1556. doi: 10.1016/j.jaci.2013.10.006 24315502

[R51] Schloss PD, Westcott SL, Ryabin T, Hall JR, Hartmann M, Hollister EB, (2009). Introducing mothur: open-source, platform-independent, community-supported software for describing and comparing microbial communities. Appl. Environ. Microbiol 75, 7537–7541. doi: 10.1128/AEM.01541-09/ASSET/91BD47E1-E1DA-4980-B8B3-5DFA9C4F1FE7/ASSETS/GRAPHIC/ZAM0230904840002.JPEG 19801464 PMC2786419

[R52] Schneeberger M, Everard A, Gómez-Valadés AG, Matamoros S, Ramírez S, Delzenne NM, (2015). Akkermansia muciniphila inversely correlates with the onset of inflammation, altered adipose tissue metabolism and metabolic disorders during obesity in mice. Sci. Rep 5, 1–14. doi: 10.1038/srep16643 PMC4643218 26563823

[R53] Schopfer FJ, Vitturi DA, Jorkasky DK, and Freeman BA (2018). Nitro-fatty acids: new drug candidates for chronic inflammatory and fibrotic diseases. Nitric. Oxide 79, 31–37. doi: 10.1016/J.NIOX.2018.06.006 29944935 PMC6280193

[R54] Sharma S, Tailor A, Warrington R, and Cheang M (2008). Is obesity associated with an increased risk for airway hyperresponsiveness and development of asthma? Allergy Asthma. Clin. Immunol 4, 51. doi: 10.1186/1710-1492-4-2-51 20525125 PMC2868882

[R55] Siegel RC, Pinnell SR, and Martin GR (1970). Cross-linking of collagen and elastin. properties of lysyl oxidase. Biochemistry 9, 4486–4492. doi: 10.1021/BI00825A004/ASSET/BI00825A004.FP.PNG_V03 5474144

[R56] Stapleton AL, Shaffer AD, Morris A, Li K, Fitch A, and Methé BA (2021). The microbiome of pediatric patients with chronic rhinosinusitis. Int. Forum Allergy Rhinol 11, 31–39. doi: 10.1002/ALR.22597 32348024

[R57] Tarabichi Y, Li K, Hu S, Nguyen C, Wang X, Elashoff D, (2015). The administration of intranasal live attenuated influenza vaccine induces changes in the nasal microbiota and nasal epithelium gene expression profiles. Microbiome 3, 74. doi: 10.1186/S40168-015-0133-2/FIGURES/7 26667497 PMC4678663

[R58] Tashiro H, Cho Y, Kasahara DI, Brand JD, Bry L, Yeliseyev V, (2019). Microbiota contribute to obesity-related increases in the pulmonary response to ozone. Am. J. Respir. Cell Mol. Biol 61, 702–712. doi: 10.1165/RCMB.2019-0144OC/SUPPL_FILE/DISCLOSURES.PDF 31144984 PMC6890400

[R59] Turnbaugh PJ, Ley RE, Mahowald MA, Magrini V, Mardis ER, and Gordon JI (2006). An obesity-associated gut microbiome with increased capacity for energy harvest. Nature 444, 1027–1031. doi: 10.1038/nature05414 17183312

[R60] Wang Q, Garrity GM, Tiedje JM, and Cole JR (2007). Naïve Bayesian classifier for rapid assignment of rRNA sequences into the new bacterial taxonomy. Appl. Environ. Microbiol 73, 5261–5267. doi: 10.1128/AEM.00062-07/SUPPL_FILE/SUMMARY_BYHIERARCHY.ZIP 17586664 PMC1950982

[R61] Wilson SJ, Ward JA, Pickett HM, Baldi S, Sousa AR, Sterk PJ, (2021). Airway elastin is increased in severe asthma and relates to proximal wall area: histological and computed tomography findings from the U-BIOPRED severe asthma study. Clin. Exp. Allergy 51, 296–304. doi: 10.1111/CEA.13813 33342006

[R62] Wu CT, Chen PJ, Lee YT, Ko JL, and Lue KH (2016). Effects of immunomodulatory supplementation with lactobacillus rhamnosus on airway inflammation in a mouse asthma model. J. Microbiol. Immunol. Infect 49, 625–635. doi: 10.1016/J.JMII.2014.08.001 25440975

[R63] Xu J, Ge J, He X, Sheng Y, Zheng S, Zhang C, (2020). Caffeic acid reduces body weight by regulating gut microbiota in diet-induced-obese mice. J. Funct. Foods 74, 104061. doi: 10.1016/J.JFF.2020.104061

[R64] Ye J, Zhao Y, Chen X, Zhou H, Yang Y, Zhang X, (2021). Pu-Erh tea ameliorates obesity and modulates gut microbiota in high fat diet fed mice. Food Res. Int 144, 110360. doi: 10.1016/J.FOODRES.2021.110360 34053553

