# Supplementary material for: Meta-omics profiling of the gut-lung axis illuminates metabolic networks and host-microbial interactions associated with elevated lung elastance in a murine model of obese allergic asthma
Source: Front Microbiomes. 2023 May 5;2:1153691. doi: 10.3389/frmbi.2023.1153691 (PMC10249466; doi:10.3389/frmbi.2023.1153691)
Supplement: Supplementary file 1 [file DataSheet_1.docx]

**Supplement Methods**

**Short Chain Fatty Acid Sample Preparation**

Short chain fatty acid sample preparation and analysis was adapted from a previously reported isotope-labeled chemical derivatization method (Han et al., 2015). Cecum samples were homogenized with 50% aqueous acetonitrile at a ratio of 15:1 vol:wt (µL:mg). Deuterated internal standards: acetate-d_4_, propionate-d_6_, and butyrate-d_5_ (5µg/mL, CDN Isotopes, Quebec, Canada) were added. Samples were homogenized using a FastPrep-24 system (MP-Bio), with Matrix D at 60 Hz for 30 sec, before being cleared of protein by centrifugation at 16,000 x g. Cleared supernatants (60µL) were collected and derivatized using 3-nitrophenylhydrazine. Each sample was mixed with 20 µL of 200 mM 3-nitrophenylhydrazine in 50% aqueous acetonitrile and 20 µL of 120 mM N-(3-dimethylaminopropyl)-N0-ethylcarbodiimide -6% pyridine solution in 50% aqueous acetonitrile. The mixture was reacted at 50ﹾC for 40 min and the reaction was stopped with 0.45 mL of 50% acetonitrile.

**Short Chain Fatty Acid Liquid Chromatography-High Resolution Mass Spectrometry (LC-HRMS) Analysis**

Derivatized samples were injected (5 µL) via a Thermo Vanquish UHPLC and separated over a reversed phase Phenomenex Kinetex C18 column (150mm x 2.1mm, 1.7µM particle size) maintained at 55°C. For the 20 min LC gradient, the mobile phase consisted of the following: solvent A (water/0.1% formic acid, FA) and solvent B (acetonitrile/0.1% FA). The gradient was the following: 0-2 min 15%B, increasing to 60%B over 10 min, increasing to 100%B over 1 min, and holding at 100%B for 3 min before equilibration at initial conditions for 4 min. The Thermo ID-X tribrid mass spectrometer was operated in positive ion mode, scanning in ddMS^2^ mode (2 μscans) from 75 to 1000 *m/z* at 120,000 resolution with an AGC target of 2e5 for full scan, and 2e4 for MS^2^ scans using HCD fragmentation at stepped 15, 35, 50 collision energies. The source ionization setting was 3.0 kV spray voltage for positive mode and source gas parameters were 45 sheath gas, 12 auxiliary gas at 320°C, and 3 sweep gas. Calibration was performed prior to analysis using the PierceTM FlexMix Ion Calibration Solutions (Thermo Fisher Scientific). Integrated peak areas were then extracted manually using Quan Browser (Thermo Fisher Xcalibur ver. 2.7). Calibration curves were created using standards and stable isotope labeled internal standards and SCFA are reported as µg per mg of cecal tissue.

**Additional Details on 16S rRNA Gene and 16S rRNA Sequencing**

Extracted genomic DNA (gDNA) and cDNA, respectively were amplified for the V4 region using Q5 HS High‐Fidelity polymerase (New England BioLabs, Ipswich, MA) with inline barcode primers design based on the method of Caporaso (Caporaso et al., 2012). V4 primer sequences were: 515f 5’-GTGCCAGCMGCCGCGGTAA-3’ and 806r 5’-GGACTACHVGGGTWTCTAAT-3’. Approximately 5-10 ng of each sample were amplified in 25 µL reactions. Cycle conditions were 98°C for 30 sec, then 30 cycles of 98°C for 10 sec, 57°C for 30 sec, and 72°C for 30 sec, with a final extension step of 72°C for 2 min. Amplicons were purified with AMPure XP beads (Beckman Coulter, Indianapolis, IN) at a 0.8:1 ratio (beads:DNA) to remove primer dimers. Eluted DNA was quantitated on a Qubit fluorimeter (Life Technologies, Grand Island, NY). Sample pooling was performed on ice by combining 40 ng of each purified band. For negative controls and poorly performing samples, 20 µL of each sample was used. The sample pool was purified with the MinElute PCR purification kit (Qiagen, Germantown, MD). The final sample pool underwent 2 more purifications: AMPure XP beads to 0.8:1 to remove primer dimers, and a final cleanup in Purelink PCR Purification Kit (Life Technologies). The purified pool was quantitated in triplicate on the Qubit fluorimeter prior to sequencing.

The sequencing pool was prepared as per Illumina’s recommendations (Illumina, Inc., San Diego, CA), with an added incubation at 95°C for 2 min immediately following the initial dilution to 20 pM. The pool was then diluted to a final concentration of 7 pM + 20% PhiX control (Illumina). Sequencing was done on an Illumina MiSeq 500‐cycle V2 kit (Illumina).

**Additional Details on 16S rRNA Gene and 16S rRNA Sequence Processing and Bioinformatics**

Sequences from the Illumina MiSeq were deconvolved and then processed through the University of Pittsburgh Center for Medicine and the Microbiome (CMM) in‐house sequence quality control pipeline, which includes dust low complexity filtering, quality value (QV<30) trimming, and trimming of primers used for 16S rRNA gene amplification, and minimum read length filtering. Using the scripts fastq_quality_trimmer and fastq_quality_filter from Hannon's Cold Spring Harbor Laboratory's FASTAX-Toolkit (http://hannonlab.cshl.edu/fastx_toolkit/). Reads were trimmed until the QV was 30 or higher. Trimmed reads shorter than 75 bp or those with less than 95% of the bases above a QV of 30 were discarded. Forward and reversed paired reads were merged with a minimum required overlap of 25 bp, proportion overlap mismatch > 0.2, maximum N's allowed = 4, and a read length minimum of 125 bp. Forward and reverse reads were merged into contigs then processed through the CMM’s Mothur‐based (v1.44.1) 16S rRNA gene sequence clustering and annotation pipeline (Schloss et al., 2009). Sequence taxonomic classifications were performed with the Ribosomal Database Project’s (RDP) naïve Bayesian classifier (Wang et al., 2007; Quast et al., 2013) with the SILVA 16S rRNA database (v138) (Quast et al., 2013).

**Supplement Results**

**Supplement Figure 1.** Treatments did not affect weights of mice in the murine model of obese allergic asthma. Mice were weighed before and after induction of allergic airway disease (AAD) with house dust mite (HDM) sensitization and challenge. AAD treatment groups included mock sensitization (adjuvant only) control (Control, n =12), AAD-Vehicle (AAD-Veh, n = 11), and AAD-NO_2_-OA (n = 11). Values are shown as mean ± SEM. Statistical testing with 2-way ANOVA and Tukey’s multiple comparisons test was performed.

**Supplement Figure 2.** Additional proinflammatory cytokines measured in the murine model of obese allergic asthma. (**A**) IL-4, (**B**) IL-5, (**C**) IL-9, (**D**) IL-12p40, and (**E**) IL-13 cytokine protein levels normalized to mg protein were measured in lung homogenates with multiplex immunoassays. (**F**) *Cxcl15* mRNA expression was measured in lung tissue with RT-qPCR normalized to *Gapdh*. Treatment groups included obese naïve (Naïve, n = 7), mock sensitization control (Control, n = 12), AAD-Vehicle (AAD-Veh, n = 11), and AAD-NO_2_-OA treatment (n =11). Values are shown as mean ± SEM. Statistical significance was calculated by ordinary one-way ANOVA with Tukey’s multiple comparisons test, *p < 0.05, **p < 0.01, ***p < 0.001, ****p < 0.0001.

**Supplement Figure 3.** Additional 16S rDNA and 16S rRNA MultiMDS plots with PERMANOVA. (**A**) 16S rDNA and (**B**) 16S rRNA MDS plots with PERMANOVA testing Cholera Toxin (CT) adjuvant as a covariate. (**C**) 16S rDNA and (**D**) 16S rRNA MDS plots with PERMANOVA testing House Dust Mite (HDM) treatment as a covariate. (**E**) 16S rDNA and (**F**) 16S rRNA MDS plots with PERMANOVA testing weight as a covariate. R^2^ and p-values from PERMANOVA calculation are reported for each plot.

**Supplement Figure 4**. Relative abundance of *Lachnospiraceae_uncl* did not differ in the 16S rRNA and 16S rDNA profiles between NO_2_-OA and Vehicle treatment. Values are shown as mean ± SEM. Statistical testing with unpaired t-test was performed.

**Supplement Figure 5**. Meta-omics intergroup distance heat map. Correlation between inter-sample distances was computed between meta-omics datasets.

**Supplement Figure 6.** Short chain fatty acids measured in obese mice with and without allergic asthma. Absolute amount (μg) of (**A**) acetate, (**B**) propionate, and (**C**) butyrate normalized to mg cecal tissue were measured with Liquid Chromatography-High Resolution Mass Spectrometry (LC-HRMS). Treatment groups included mock sensitization control (Control, n = 4), AAD-Vehicle (AAD-Veh, n = 12), and AAD-NO_2_-OA (n =11). Values are shown as mean ± SEM. Statistical testing with ordinary one-way ANOVA and Tukey’s multiple comparisons test was performed.

**Supplement Table 1: Significant associations between measured variables in the Treatment-Measured-Response (TMR) Model**

| **Link #** | **Model Type** | **Model (Predictor 🡪 Response)** | **Predictor** | **Response** | **β** | **p-value** |
| --- | --- | --- | --- | --- | --- | --- |
| 1 | Measured to Measured | 16S DNA Profile Taxa−>16S RNA Profile Taxa | Total Weight Gain (log) | *Lactobacillus* (RNA Profiles) | 8.437 | 3.12e−03 |
| 2 | Measured to Measured | 16S DNA Profile Taxa−>Lung metabolites | *Dubosiella* (DNA Profiles) | Lung Hydroxyproline (log) | −0.304 | 8.37e−04 |
| 3 | Measured to Measured | 16S DNA Profile Taxa−>Lung metabolites | *Lactobacillus* (DNA Profiles) | Lung Hydroxyproline (log) | 0.182 | 2.05e−03 |
| 4 | Measured to Measured | 16S DNA Profile Taxa−>Lung metabolites | Total Weight Gain (log) | Lung Hydroxyproline (log) | −3.995 | 2.17e−03 |
| 5 | Measured to Measured | 16S DNA Profile Taxa−>Lung metabolites | Final Weight | Lung Hydroxyproline (log) | 0.116 | 2.71e−03 |
| 6 | Measured to Measured | 16S DNA Profile Taxa−>Serum metabolites | *Dubosiella* (DNA Profiles) | Serum Alanine | 0.949 | 1.73e−03 |
| 7 | Measured to Measured | 16S DNA Profile Taxa−>Serum metabolites | *Lactobacillus* (DNA Profiles) | Serum Alanine | −0.623 | 1.99e−03 |
| 8 | Measured to Measured | 16S RNA Profile Taxa−>Cecum metabolites | NO_2_-OA Treatment | Cecum Hydroxyproline (log) | 0.441 | 3.67e−03 |
| 9 | Measured to Measured | 16S RNA Profile Taxa−>Cecum metabolites | *Lachnoclostridium* (RNA Profiles) | Cecum Hydroxyproline (log) | 0.363 | 1.09e−03 |
| 10 | Covariate to Measured | Cecum metabolites | CT adjuvant | Cecum Glycine | 0.054 | 1.03e−03 |
| 11 | Covariate to Measured | Cecum metabolites | CT adjuvant | Cecum Glutamine (Sqrt) | 0.581 | 9.13e−04 |
| 12 | Measured to Measured | Cecum metabolites−>16S DNA Profile Taxa | NO_2_-OA Treatment | *Muribaculaceae_ge* (DNA Profiles) | 2.345 | 4.23e−03 |
| 13 | Measured to Measured | Cecum metabolites−>16S RNA Profile Taxa | Cecum Valine (log) | *Lachnoclostridium* (RNA Profiles) | −3.587 | 2.29e−03 |
| 14 | Measured to Measured | Cecum metabolites−>16S RNA Profile Taxa | Cecum Valine (log) | *Lachnospiraceae_uncl* (RNA Profiles) | −3.117 | 1.81e−03 |
| 15 | Measured to Measured | Cecum metabolites−>16S RNA Profile Taxa | Cecum Valine (log) | *Lachnospiraceae_uncl* (RNA Profiles)trd | −3.646 | 4.15e−03 |
| 16 | Measured to Measured | Cecum metabolites−>16S RNA Profile Taxa | Cecum Cytidine (log) | *Oscillibacter* (RNA Profiles) | −1.711 | 1.25e−03 |
| 17 | Measured to Measured | Cecum metabolites−>16S RNA Profile Taxa | Cecum Valine (log) | *Oscillibacter* (RNA Profiles) | −2.598 | 3.77e−03 |
| 18 | Measured to Measured | Cecum metabolites−>16S RNA Profile Taxa | Cecum Glycine | *Oscillospiraceae_Uncltrd* (RNA Profiles) | 40.759 | 4.49e−03 |
| 19 | Measured to Measured | Cecum metabolites−>16S RNA Profile Taxa | Cecum Cytidine (log) | *Oscillospiraceae_Uncltrd* (RNA Profiles) | −1.785 | 2.55e−03 |
| 20 | Measured to Measured | Colon mRNA expression−>Cecum metabolites | CT adjuvant | Cecum Glycine | 0.0544 | 2.19e−03 |
| 21 | Measured to Measured | Colon mRNA expression−>Cecum metabolites | CT adjuvant | Cecum Glutamine (Sqrt) | 0.557 | 4.67e−03 |
| 22 | Measured to Measured | Colon mRNA expression−>Lung metabolites | Colon Cox2 mRNA Expression | Lung Cisaconitate | −2.959 | 1.80e−03 |
| 23 | Measured to Measured | Colon mRNA expression−>Lung metabolites | log_Nos2_C | Lung Cisaconitate | 1.275 | 1.94e−03 |
| 24 | Measured to Measured | Lung metabolites−>Serum metabolites | Lung Glutamate | Serum Alanine | −0.043 | 4.41e−03 |
| 25 | Measured to Measured | Lung metabolites−>Serum metabolites | Lung Hydroxyproline (log) | Serum Alanine | −2.771 | 6.79e−04 |
| 26 | Measured to Measured | Lung metabolites−>Serum metabolites | Lung Glutamate | Serum Asparagine | −0.008 | 2.26e−04 |
| 27 | Measured to Measured | Lung metabolites−>Stool metabolites | Lung Cisaconitate | Stool Proline (log) | 0.136 | 3.19e−03 |
| 28 | Measured to Measured | Lung metabolites−>Stool metabolites | Lung Cisaconitate | Stool Valine (log) | 0.096 | 4.15e−04 |
| 29 | Measured to Measured | Lung metabolites−>Stool metabolites | Final Weight | Stool Valine (log) | −0.087 | 2.66e−03 |
| 30 | Measured to Measured | Lung metabolites−>Stool metabolites | Lung Cisaconitate | Stool Glutamine (Sqrt) | 0.099 | 2.29e−03 |
| 31 | Measured to Measured | Lung mRNA expression−>16S DNA Profile Taxa | Lung Muc5b mRNA Expression (log) | *Lactobacillus* (DNA Profiles) | −1.996 | 8.77e−04 |
| 32 | Measured to Measured | Lung mRNA expression−>16S DNA Profile Taxa | NO2-OA Treatment | *Lactobacillus* (DNA Profiles) | 3.592 | 1.15e−03 |
| 33 | Measured to Measured | Lung mRNA expression−>16S DNA Profile Taxa | Lung Muc5b mRNA Expression (log) | *Romboutsia* (DNA Profiles) | −1.299 | 2.71e−03 |
| 34 | Measured to Measured | Lung mRNA expression−>Serum metabolites | Lung Nos2 mRNA Expression (log) | Serum Histidine | −2.275 | 3.52e−03 |
| 35 | Measured to Measured | Serum metabolites−>Colon mRNA expression | Serum Alanine | Colon Cox2 mRNA Expression | −0.448 | 1.07e−03 |
| 36 | Measured to Measured | Serum metabolites−>Lung metabolites | Serum Alanine | Lung Hydroxyproline (log) | −0.192 | 1.55e−03 |
| 37 | Measured to Measured | Stool Metabolites−>16S DNA Profile Taxa | Stool Glutamine (Sqrt) | *Bacteroides* (DNA Profiles) | 2.346 | 4.36e−03 |
| 38 | Measured to Measured | Stool Metabolites−>16S DNA Profile Taxa | Stool Proline (log) | *Lachnoclostridium* (DNA Profiles) | −3.749 | 1.35e−03 |
| 39 | Measured to Measured | Stool Metabolites−>16S DNA Profile Taxa | Stool Serine (log) | *Lachnoclostridium* (DNA Profiles) | 4.442 | 7.33e−04 |
| 40 | Measured to Measured | Stool Metabolites−>16S DNA Profile Taxa | Stool Proline (log) | *Lachnospiraceae_uncl* (DNA Profiles) | −3.661 | 2.53e−04 |
| 41 | Measured to Measured | Stool Metabolites−>16S DNA Profile Taxa | Stool Serine (log) | *Lachnospiraceae_uncl* (DNA Profiles) | 4.296 | 1.36e−04 |
| 42 | Measured to Measured | Stool Metabolites−>16S DNA Profile Taxa | Stool Valine (log) | *Lactobacillus* (DNA Profiles) | 6.617 | 1.53e−04 |
| 43 | Measured to Measured | Stool Metabolites−>16S DNA Profile Taxa | Final Weight | *Lactobacillus* (DNA Profiles) | 0.418 | 1.45e−03 |
| 44 | Measured to Measured | Stool Metabolites−>16S DNA Profile Taxa | Stool Proline (log) | *Muribaculaceae_ge* (DNA Profiles) | −4.420 | 3.18e−03 |
| 45 | Measured to Measured | Stool Metabolites−>16S DNA Profile Taxa | Stool Serine (log) | *Muribaculaceae_ge* (DNA Profiles) | 5.551 | 1.13e−03 |
| 46 | Measured to Measured | Stool Metabolites−>16S DNA Profile Taxa | CT adjuvant | *Oscillospiraceae_Uncltrd* (DNA Profiles) | 3.242 | 3.03e−03 |
| 47 | Measured to Measured | Stool Metabolites−>16S DNA Profile Taxa | Stool Serine (log) | *Oscillospiraceae_Uncltrd* (DNA Profiles) | 3.993 | 4.47e−03 |
| 48 | Measured to Measured | Stool Metabolites−>16S DNA Profile Taxa | Total Weight Gain (log) | *Oscillospiraceae_Uncltrd* (DNA Profiles) | −10.31 | 2.31e−03 |
| 49 | Measured to Measured | Stool Metabolites−>16S DNA Profile Taxa | Final Weight | *Oscillospiraceae_Uncltrd* (DNA Profiles) | 0.303 | 3.83e−03 |
| 50 | Measured to Measured | Stool Metabolites−>16S DNA Profile Taxa | CT adjuvant | *Romboutsia* (DNA Profiles) | 3.189 | 1.76e−04 |
| 51 | Measured to Measured | Stool Metabolites−>16S DNA Profile Taxa | HDM Sensitization | *Romboutsia* (DNA Profiles) | 3.879 | 1.50e−04 |
| 52 | Measured to Measured | Stool Metabolites−>16S DNA Profile Taxa | Stool Serine (log) | *Romboutsia* (DNA Profiles) | 5.087 | 1.76e−05 |
| 53 | Measured to Measured | Stool Metabolites−>16S DNA Profile Taxa | Stool Glutamine (Sqrt) | *Romboutsia* (DNA Profiles) | −2.095 | 4.38e−03 |
| 54 | Measured to Measured | Stool Metabolites−>16S RNA Profile Taxa | HDM Sensitization | *Bacteroides* (RNA Profiles) | −2.661 | 3.96e−04 |
| 55 | Measured to Measured | Stool Metabolites−>16S RNA Profile Taxa | Stool Serine (log) | *Dubosiella* (RNA Profiles) | 4.723 | 3.64e−03 |
| 56 | Measured to Measured | Stool Metabolites−>16S RNA Profile Taxa | Stool Proline (log) | *Lachnoclostridium* (RNA Profiles) | −4.190 | 2.88e−04 |
| 57 | Measured to Measured | Stool Metabolites−>16S RNA Profile Taxa | Stool Serine (log) | *Lachnoclostridium* (RNA Profiles) | 4.700 | 2.46e−04 |
| 58 | Measured to Measured | Stool Metabolites−>16S RNA Profile Taxa | Stool Proline (log) | *Lachnospiraceae_uncl* (RNA Profiles) | −3.381 | 2.06e−03 |
| 59 | Measured to Measured | Stool Metabolites−>16S RNA Profile Taxa | Stool Serine (log) | *Lachnospiraceae_uncl* (RNA Profiles) | 3.968 | 1.25e−03 |
| 60 | Measured to Measured | Stool Metabolites−>16S RNA Profile Taxa | Stool Serine (log) | *Lachnospiraceae_uncltrd* (RNA Profiles) | 4.649 | 3.00e−04 |
| 61 | Measured to Measured | Stool Metabolites−>16S RNA Profile Taxa | Final Weight | *Lachnospiraceae_uncltrd* (RNA Profiles) | 0.279 | 2.02e−03 |
| 62 | Measured to Measured | Stool Metabolites−>16S RNA Profile Taxa | Stool Serine (log) | *Lactobacillus* (RNA Profiles) | 5.928 | 2.73e−03 |
| 63 | Measured to Measured | Stool Metabolites−>16S RNA Profile Taxa | Stool Proline (log) | *Oscillibacter* (RNA Profiles) | −3.767 | 8.50e−05 |
| 64 | Measured to Measured | Stool Metabolites−>16S RNA Profile Taxa | Stool Serine (log) | *Oscillibacter* (RNA Profiles) | 4.157 | 8.57e−05 |
| 65 | Measured to Measured | Stool Metabolites−>16S RNA Profile Taxa | Stool Proline (log) | *Oscillospiraceae_Uncltrd* (RNA Profiles) | −3.441 | 1.09e−03 |
| 66 | Measured to Measured | Stool Metabolites−>16S RNA Profile Taxa | Stool Serine (log) | *Oscillospiraceae_Uncltrd* (RNA Profiles) | 3.784 | 1.13e−03 |
| 67 | Measured to Measured | Stool Metabolites−>16S RNA Profile Taxa | Stool Serine (log) | *Romboutsia* (RNA Profiles) | 4.081 | 2.19e−03 |
| 68 | Measured to Measured | Stool Metabolites−>Serum metabolites | Total Weight Gain (log) | Serum Hydroxyproline | −1.028 | 3.12e−03 |
| 69 | Measured to Measured | Stool Metabolites−>Serum metabolites | Final Weight | Serum Hydroxyproline | 0.042 | 3.46e−04 |

**Supplement References**

Caporaso, J. G., Lauber, C. L., Walters, W. A., Berg-Lyons, D., Huntley, J., Fierer, N., et al. (2012). Ultra-high-throughput microbial community analysis on the Illumina HiSeq and MiSeq platforms. *ISME J. 2012 68* 6, 1621–1624. doi: 10.1038/ismej.2012.8.

Han, J., Lin, K., Sequeira, C., and Borchers, C. H. (2015). An isotope-labeled chemical derivatization method for the quantitation of short-chain fatty acids in human feces by liquid chromatography–tandem mass spectrometry. *Anal. Chim. Acta* 854, 86–94. doi: 10.1016/J.ACA.2014.11.015.

Quast, C., Pruesse, E., Yilmaz, P., Gerken, J., Schweer, T., Yarza, P., et al. (2013). The SILVA ribosomal RNA gene database project: improved data processing and web-based tools. *Nucleic Acids Res.* 41, D590. doi: 10.1093/NAR/GKS1219.

Schloss, P. D., Westcott, S. L., Ryabin, T., Hall, J. R., Hartmann, M., Hollister, E. B., et al. (2009). Introducing mothur: Open-source, platform-independent, community-supported software for describing and comparing microbial communities. *Appl. Environ. Microbiol.* 75, 7537–7541. doi: 10.1128/AEM.01541-09/ASSET/91BD47E1-E1DA-4980-B8B3-5DFA9C4F1FE7/ASSETS/GRAPHIC/ZAM0230904840002.JPEG.

Wang, Q., Garrity, G. M., Tiedje, J. M., and Cole, J. R. (2007). Naïve Bayesian classifier for rapid assignment of rRNA sequences into the new bacterial taxonomy. *Appl. Environ. Microbiol.* 73, 5261–5267. doi: 10.1128/AEM.00062-07/SUPPL_FILE/SUMMARY_BYHIERARCHY.ZIP.
